# Cellular sequestrases maintain basal Hsp70 capacity ensuring balanced proteostasis

**DOI:** 10.1038/s41467-019-12868-1

**Published:** 2019-10-24

**Authors:** Chi-ting Ho, Tomas Grousl, Oren Shatz, Areeb Jawed, Carmen Ruger-Herreros, Marije Semmelink, Regina Zahn, Karsten Richter, Bernd Bukau, Axel Mogk

**Affiliations:** 1 Center for Molecular Biology of Heidelberg University (ZMBH), Im Neuenheimer Feld 282, DKFZ-ZMBH Alliance, Heidelberg, Germany; 2 German Cancer Research Center (DKFZ), 69120 Heidelberg, Germany; 3 Present Address: Institute of Microbiology of the Czech Academy of Sciences, Prague, Czech Republic

**Keywords:** Biochemistry, Chaperones, Protein aggregation

## Abstract

Maintenance of cellular proteostasis is achieved by a multi-layered quality control network, which counteracts the accumulation of misfolded proteins by refolding and degradation pathways. The organized sequestration of misfolded proteins, actively promoted by cellular sequestrases, represents a third strategy of quality control. Here we determine the role of sequestration within the proteostasis network in *Saccharomyces cerevisiae* and the mechanism by which it occurs. The Hsp42 and Btn2 sequestrases are functionally intertwined with the refolding activity of the Hsp70 system. Sequestration of misfolded proteins by Hsp42 and Btn2 prevents proteostasis collapse and viability loss in cells with limited Hsp70 capacity, likely by shielding Hsp70 from misfolded protein overload. Btn2 has chaperone and sequestrase activity and shares features with small heat shock proteins. During stress recovery Btn2 recruits the Hsp70-Hsp104 disaggregase by directly interacting with the Hsp70 co-chaperone Sis1, thereby shunting sequestered proteins to the refolding pathway.

## Abstract (web summary)

The sequestration of misfolded proteins into large assemblies by sequestrases is now considered as the third pillar in protein quality control besides chaperones and proteases. Here the authors characterise the functions of the sequestrases Hsp42 and Btn2 in the proteostasis network of *S. cerevisiae* and find that they protect cells from too exhaustive depletion of the Hsp70 system.

## Introduction

The cellular network that maintains protein homeostasis (proteostasis) has long been considered to rely on two main strategies: to promote the refolding of misfolded proteins by molecular chaperones and to degrade them by proteolytic systems. The cellular capacity to pursue these strategies, however, becomes limited under severe protein folding stress and declines during aging, ultimately resulting in increased protein aggregation^1–4^. The accumulation of aggregates has therefore been viewed as a hallmark of proteostasis collapse, and aggregation as uncontrolled process of denatured proteins that clump together via hydrophobic interactions.

This view has recently changed due to several lines of observations. The aggregation of proteins under severe heat stress is not random, but mainly involves proteins that function in protein synthesis (e.g. translation), and hence constitutes a cellular response to control the flux of newly synthesized proteins into the proteome^5,6^. Misfolded proteins are deposited at inclusions located to specific cellular sites^7–9^, through a process that depends on dedicated molecular chaperones, termed sequestrases^8,10–15^. Hence, “protein aggregation” under these conditions is the result of a regulated cellular process, and represents an organized sequestration event.

In *Saccharomyces cerevisiae* Hsp42 and Btn2 promote protein sequestration during moderate heat stress, genotoxic stress and cellular aging^11–17^. Hsp42 and Btn2 act largely compartment-specific by controlling sequestrations in the cytosol (CytoQ, Q-bodies) and nucleus (INQ), respectively. Both chaperones additionally undergo inter-compartmental cross-talk, affecting protein sequestration in the other compartment^13,17^. Hsp42 is a member of the small heat-shock protein (sHsp) family and harbours a disordered prion-like domain (PrLD) that is essential for sequestrase function^18^. In contrast, Btn2 is largely uncharacterized.

The sequestration of misfolded proteins is now recognized as the third strategy of the proteostasis network, helping cells to cope with an overload of misfolded proteins^19–21^. But how important sequestrases exactly are in relation to the two other proteostasis strategies, and to what extent they contribute to the buffering of cytotoxicity induced by misfolding has remained elusive. Several beneficial consequences of sequestrase activities have been suggested. First, sequestration of misfolded proteins confines their accessible sticky surface, thereby eventually reducing cytotoxicity^1,22–26^. This might also, secondly, prevent exhaustion of finite resources of chaperone and protease systems. Third, the formation of protein sequestrations can facilitate asymmetric inheritance of damaged proteins, allowing for formation of aggregate-free daughter cells^27–29^. Fourth, the spatial concentration of misfolded proteins through sequestration might aid chaperone and proteolytic activities to facilitate repair or clearance^30^. Whether and how sequestrases target sequestered substrates to refolding or proteolytic pathways is unknown. A major limitation in analysing sequestrase function experimentally has been the absence of strong growth phenotypes of respective mutant cells.

Here we dissect the function and mechanism of Hsp42- and Btn2-mediated, organized protein sequestration for stress biology of yeast cells. We show that the Hsp42 and Btn2 become essential for cell growth upon confining Hsp70 capacity, by ensuring basal Hsp70 activity and preventing proteostasis collapse. We biochemically define Btn2 domains that execute distinct functions in both protein sequestration during stress and recruiting Hsp70/Hsp100 disaggregases for refolding of sequestered proteins during stress recovery. Our findings imply that the two sequestrases constitute a stress rescue system that is essential for viability upon misfolded protein overload, by mitigating the burden on the Hsp70 chaperone machinery.

## Results

### Sequestrases become crucial in cells with low Hsp70 capacity

Btn2 and Hsp42 organize protein sequestration in *S. cerevisiae* cells. However, respective knockout cells do not show pronounced growth defects under stress conditions^13^. We hypothesized that the absence of a strong phenotype results from compensatory activities of other proteostasis components involved in protein folding or degradation pathways, superseding the need for sequestrases. Accordingly, sequestrases might become important in cells with reduced proteolytic or refolding capacities. We therefore screened for synthetic sickness of *btn2Δ* and *hsp42Δ* cells in mutant backgrounds with reduced proteasome or chaperone activities.

To lower proteasome activity we used *rpn4Δ* and *irc25Δ* knockouts, which are compromised in expression of proteasomal genes and 26S proteasome assembly, respectively^31,32^. The mutations result in reduced levels of 26S proteasomes and temperature-sensitive growth^33^ (Supplementary Fig. 1a). Additionally, we employed *rsp5-3* and *ubr1Δ/san1Δ* mutants, which lack E3 ligases that play key roles in the ubiquitination and degradation of misfolded proteins^34–37^. We did not observe increased temperature sensitivity when deleting *btn2* or *hsp42* in these mutants, suggesting that degradation and sequestration of heat-induced misfolded proteins are not interlocked in a phenotypically apparent manner (Supplementary Fig. 1a).

**Fig. 1:**
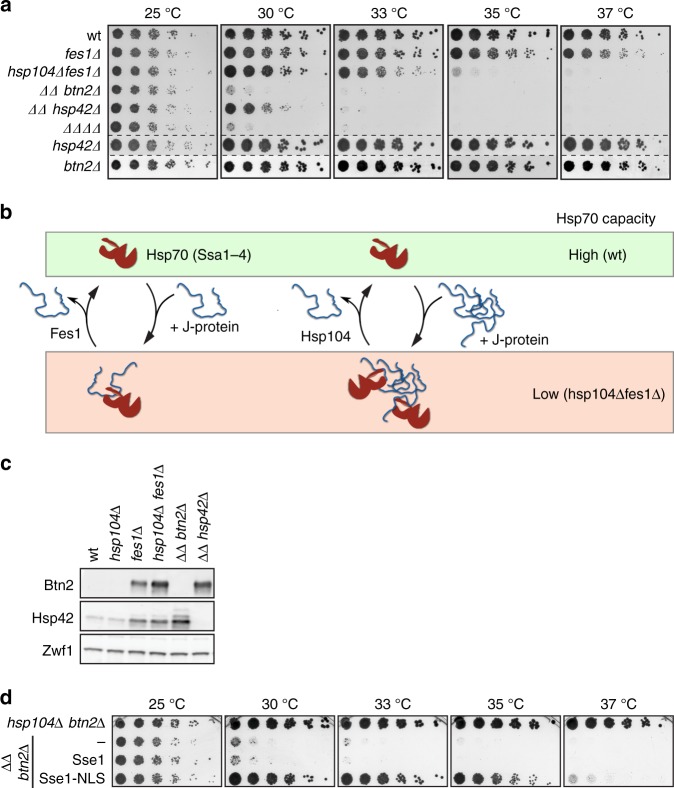
Btn2 becomes essential for cell growth upon confining nuclear Hsp70 capacity. **a** Serial dilutions of indicated *S. cerevisiae* wild type (wt) and chaperone mutant cells (*ΔΔ*: *hsp104Δ fes1Δ*; *ΔΔΔΔ*: *hsp104Δ fes1Δ btn2Δ hsp42Δ*) were spotted on YPD plates and incubated at indicated temperatures for 2 days. **b** The nucleotide exchange factor Fes1 and the Hsp104 disaggregase displace Hsp70-bound substrates, thereby recycling Hsp70 and ensuring high Hsp70 capacity (wt cells). Lack of these Hsp70 co-chaperones in *hsp104Δ fes1Δ* cells cause low Hsp70 capacity. **c** Btn2 and Hsp42 levels were determined at 30 °C in *S. cerevisiae* wt and chaperone mutant cells by western blot analysis. Zwf1 levels were determined as a loading control. **d** Serial dilutions of indicated *S. cerevisiae* chaperone mutant cells were spotted on YPD plates and incubated at indicated temperatures for 2 days. *ΔΔ btn2Δ* cells expressed an additional copy of either Sse1 or Sse1-NLS, harbouring a C-terminal nuclear localization sequence

We next tested for an interconnection between Btn2/Hsp42 and the Hsp70 system, which is a central component of the proteostasis network^38,39^. We employed several strategies to reduce the capacity of the four cytosolic/nuclear Hsp70 chaperones, Ssa1-4, which collectively are essential (Fig. 1a, Supplementary Fig. 1b). First, we deleted the *ssa1* and *ssa2* genes encoding for the two major Ssa’s, but did not observe synthetic sickness when linking *ssa1Δ ssa2Δ* to *btn2Δ* or *hsp42Δ* (Supplementary Fig. 1b). Second, we employed deletions of genes encoding the Hsp70 partner chaperones Sse1, Fes1 and Hsp104. Fes1 and Sse1 function as nucleotide exchange factors (NEFs) of Ssa’s, catalysing ADP to ATP exchange, which promotes dissociation of bound substrates^40^. The AAA + chaperone Hsp104 cooperates with Hsp70 in protein disaggregation, taking over Ssa-bound substrates through direct Ssa-Hsp104 interaction^41,42^. In the respective mutant cells, Ssa-substrate interactions will therefore be stabilized, reducing the available pool of cytosolic/nuclear Hsp70 chaperones, and hence the cellular Hsp70 capacity (Fig. 1b). Third, we depleted the essential J-protein Sis1 in the presence and absence of Hsp42 and Btn2. Depletion of Sis1 will lower Hsp70 activity and hamper Hsp70-dependent folding processes.

We observed severe synthetic growth defects at 30 °C when deleting *btn2* in *fes1Δ hsp104Δ* (also referred to as *ΔΔ*) mutant cells, but not in other mutant combinations (i.e. *sse1Δ fes1Δ* and *sse1Δ hsp104Δ*) (Fig. 1a, Supplementary Fig. 1b). Deleting *hsp42* in *sse1Δ hsp104Δ* mutants also caused a temperature-sensitive growth phenotype at 37 °C (Supplementary Fig. 1b). Furthermore, lowering Sis1 levels by placing it under the control of a doxycycline-repressible promoter caused synthetic sickness at high temperatures (37 °C/39 °C) when combined with *btn2Δ* and *hsp42Δ* knockouts (Supplementary Fig. 1d, e). Altogether, these findings manifest that sequestrases become crucial in cells with low Hsp70 activity. Since *ΔΔ btn2Δ* cells exhibited the strongest growth deficiencies, we focussed our analysis on those cells.

At 33 °C we also observed synthetic sickness when deleting *hsp42* in *ΔΔ* cells (Fig. 1a). A quadruple knockout *ΔΔ btn2Δ hsp42Δ* (also referred to as *∆∆∆∆*) did not increase temperature sensitivity further. This suggests a dominant function of Btn2 in cell protection under Hsp70-limited conditions. Synthetic sickness of *ΔΔ btn2Δ, ΔΔ hsp42Δ* and *ΔΔ btn2Δ hsp42Δ* was confirmed when growth curves of respective cells were recorded at different temperatures in liquid media (Supplementary Fig. 1c).

Why do *btn2Δ* cells specifically show a strong genetic interaction with *fes1Δ hsp104Δ* (*ΔΔ*) cells? *ΔΔ* cells exhibit the strongest temperature sensitivity when compared to all other Hsp70 mutant strains tested, suggesting that Hsp70 capacity is particularly low in these cells (Supplementary Fig. 1b). Btn2 levels were particularly high in *ΔΔ* cells, consistent with high activity of the heat-shock transcription factor Hsf1 (which controls *btn2* expression) resulting from low Hsp70 activity, which negatively regulates Hsf1 function (Fig. 1c, Supplementary Fig. 2a)^43,44^. In line with an HSF1-mediated heat-shock response, the levels of Hsp42 (Fig. 1c) and other chaperones (e.g. Ssa1-4, Hsp26) (Supplementary Fig. 2b) were high in *ΔΔ* and further increased (Hsp42, Hsp26) in *ΔΔ btn2Δ* cells. We directly demonstrate activation of the heat-shock response in *ΔΔ* cells by use of a *gfp* reporter gene placed under control of Hsf1 (Supplementary Fig. 2c). This automatic increase of Btn2 but also Hsp42 levels in the Hsp70 mutant background explains how the sequestrases can increase their capacity and thereby act as efficient rescue system to maintain cell growth. Notably, the increased expression of other Hsf1-dependent chaperones does not rescue the growth defect of *ΔΔ btn2Δ* cells, documenting that *ΔΔ* cells are particularly dependent on sequestrase activity.

An increased temperature sensitivity was not observed when introducing the *btn2Δ* allele in single *fes1Δ* or *hsp104Δ* mutant cells, although *fes1Δ btn2Δ* cells formed smaller colonies as compared to respective single knockouts, indicating minor synthetic growth defects (Supplementary Fig. 2d). We also introduced the *hsp26Δ* allele into *ΔΔ* cells. Hsp26 is the second bona fide yeast sHsp, which, however, requires activation by heat stress and does not exhibit sequestrase activity^11,45^. We did not observe increased temperature sensitivity for*ΔΔ hsp26Δ* cells (Supplementary Fig. 2d). The Hsp90 chaperone system represents another major chaperone system of yeast cells that is essential for viability. When lowering Hsp90 activity upon addition of the Hsp90-specific inhibitor Radicicol, no synthetic growth defects were observed in *btn2Δ* cells (Supplementary Fig. 2e). Together, these findings indicate a functional interconnection specifically between the Hsp70 chaperone system and the sequestrases Btn2 and Hsp42.

To further support an essential function of Btn2 for cell growth at reduced Hsp70 capacity, we sought to rescue growth of *ΔΔ btn2Δ* cells by re-increasing Hsp70 availability. First, we overproduced Ssa1 from plasmid p415GPD-Ssa1, increasing total Hsp70 levels by 2-fold; this only partially rescued growth of *ΔΔ btn2Δ* cells at 33 °C (Supplementary Fig. 3a, b). The weak complementation can be explained by the specific defect of *ΔΔ* cells in releasing the substrate from Hsp70, which is not overcome by Ssa1 overproduction.

To specifically rescue this deficiency of *ΔΔ* cells, we targeted Sse1, the major NEF for Ssa’s^46,47^ to the nucleus. Sse1 is largely excluded from the nucleus, whereas Fes1 is located in both the cytosol and the nucleus (Supplementary Fig. 3c)^48,49^. *ΔΔ* cells therefore lack the sole nuclear NEF, rationalizing growth dependence on nuclear Btn2. We expressed Sse1-NLS harbouring a C-terminal nuclear localization sequence (NLS) as additional copy at low levels in *ΔΔ btn2Δ* cells (Fig. 1d, Supplementary Fig. 3d). Sse1-NLS expression fully restored growth of *ΔΔ btn2Δ* cells up to 35 °C, demonstrating that re-increasing nuclear Hsp70 capacity overcomes growth dependence on Btn2. An NLS-tagged Sse1 mutant that harbours multiple mutations in its substrate binding site (Sse1_SBD_: L433A/N434P/F439/M441A) and is strongly affected in substrate binding^50^ rescued growth of *ΔΔ btn2Δ* cells at 30 °C, supporting that NEF activity of Sse1 is most important (Supplementary Fig. 3e). Complementation activity of Sse1_SBD_ was reduced at increased temperatures, suggesting that Sse1 chaperone activity also contributes to growth rescue in agreement with former findings^50^.

### Lack of sequestrase activities causes proteostasis collapse

**Fig. 2:**
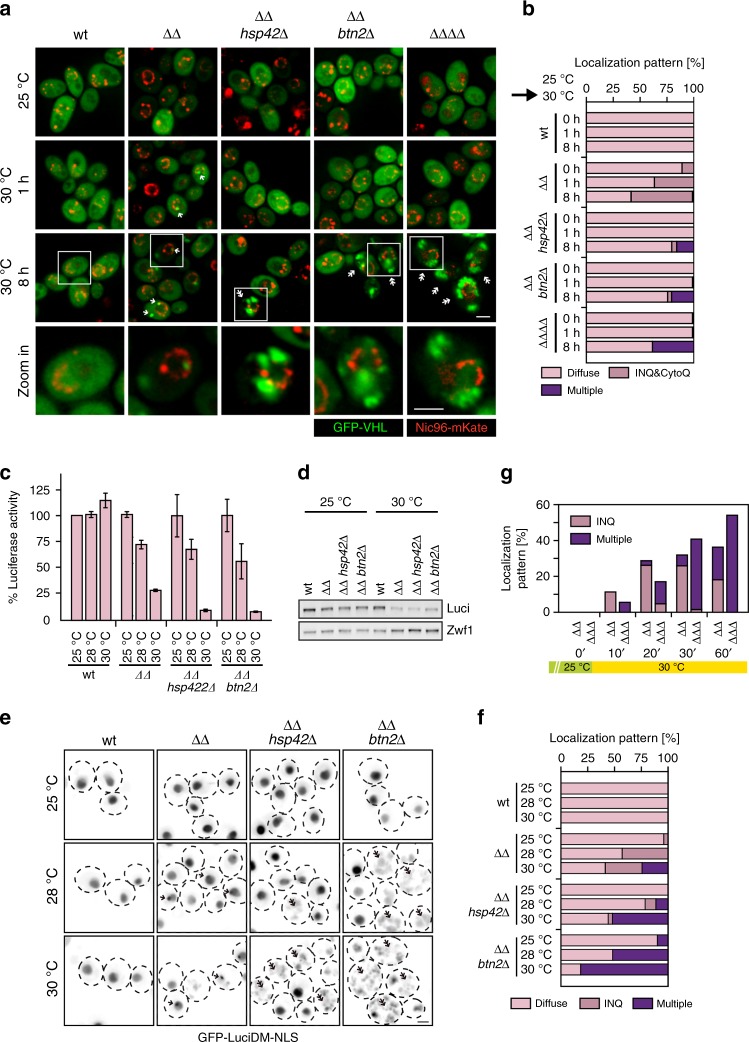
Loss of Btn2 causes proteostasis collapse. **a**, **b ***Saccharomyces cerevisiae* wild type (wt) and indicated chaperone mutant cell (*ΔΔ*: *hsp104Δ fes1Δ*; *ΔΔΔΔ*: *hsp104Δ fes1Δ btn2Δ hsp42Δ*) expressing the misfolded reporter GFP-VHL were grown at 25 °C to mid-exponential growth phase and shifted to 30 °C for 1 and 8 h. The cellular localization of GFP-VHL was determined and quantified (*n* > 100, 2 replicates). The nuclear envelope was labelled by coexpression of fluorescent Nic96-mKate2 nucleoporin. INQ (1 GFP-VHL foci) and CytoQ (1–2 GFP-VHL foci) are located inside and outside the nucleus (single arrow). Massive GFP-VHL aggregation leads to the formation of multiple (*n* > 3) GFP-VHL foci (double arrow). **c**–**f** *Saccharomyces cerevisiae* wild type (wt) and indicated chaperone mutant cells expressing GFP-LuciDM-NLS were grown in SC medium at indicated temperatures. Luciferase activities (**c**), levels (**d**) and cellular localizations (**e**, **f**) were determined. Luciferase activities were normalized to cell numbers and the activity determined in wt cells at 25 °C was set to 100% (**c**). Standard deviations were calculated based on three independent experiments. Levels of GFP-LuciDM-NLS were determined by western blot analysis. A Zwf1 loading control is provided (**d**). GFP-LuciDM-NLS localizations were quantified for *n* = 100 cells (2 replicates). INQ foci (single arrow) are located at the rim of the nucleus stained by diffuse GFP-LuciDM-NLS and cells suffering from proteostasis collapse (multiple GFP-LuciDM-NLS foci) are marked by double arrow (**e**, **f**). **g** *Saccharomyces cerevisiae hsp104Δ fes1Δ* (*ΔΔ*) and *hsp104Δ fes1Δ btn2Δ* (*ΔΔΔ*) cells expressing GFP-LuciDM-NLS were grown at 25 °C and shifted to 30 °C. Cellular localizations of GFP-LuciDM-NLS were monitored and the fraction of cells showing single INQ and multiple (*n* > 3) cytosolic foci were determined at the indicated time points (*n* = 50, 2 replicates)

We set out to directly link the determined growth deficiencies of *ΔΔ btn2Δ* and *ΔΔ hsp42Δ* cells to alterations in proteostasis. To monitor protein sequestration patterns in these mutants, we tested the intracellular localization of the constitutively misfolded reporter GFP-VHL^7^. As compared to wild type (wt), the levels of GFP-VHL were increased to similar degrees in all mutant cells tested *(∆∆*, *∆∆ btn2∆*, *∆∆ hsp42∆*, *∆∆∆∆*} (Supplementary Fig. 3f), consistent with the previously reported role of the Hsp70 system in the degradation of soluble VHL^51^ and the limited Hsp70 capacity in the mutants. At 25 °C, GFP-VHL showed diffuse fluorescence in wt, but formed nuclear foci (INQ) in a subset (11%) of *ΔΔ* cells (Fig. 2a, b). GFP-VHL foci were not observed in *ΔΔ btn2Δ* and *ΔΔ hsp42Δ* cells, indicating that foci formation results from an active, Hsp42- or Btn2-mediated sequestration process and not from a general proteostasis collapse. Co-localization of Btn2 and GFP-VHL foci in *ΔΔ* cells confirmed a direct role of Btn2 for the sequestration (Supplementary Fig. 3g). A puzzling finding is the absence of nuclear INQ formation in *ΔΔ hsp42Δ* cells (Fig. 2a). This suggests that the activities of cytosolic and nuclear sequestrases, Hsp42 and Btn2, respectively, can be interdependent in agreement with former findings^17,52^. The basis of this relationship is currently unknown and the interdependence as described here was not observed earlier when applying more severe proteotoxic stress^13^.

We next monitored the short- and long-term consequences of preventing organized protein sequestration in *∆∆ btn2Δ* and *∆∆ hsp42Δ* cells by exposing the cells to non-permissive growth conditions (30 °C). Shifting *∆∆* cells from 25°C to 30°C for 60 min increased the fraction of cells harbouring nuclear GFP-VHL foci (INQ) from 11 to 32% and this number further increased to 49% upon 8-h incubation (Fig. 2a, b); *∆∆* cells additionally harboured 1–2 cytosolic GFP-VHL foci (CytoQ) (60 min: 15%, 8 h: 29%). At the initial phase (60 min) of temperature upshift, GFP-VHL sequestration was not observed in *∆∆* cells, additionally lacking either Btn2 or Hsp42 (Fig. 2a, b). Prolonged incubation at non-permissive conditions, however, caused splitting of *∆∆ btn2Δ*, *∆Δ hsp42Δ* and *∆Δ hsp42Δ btn2Δ* cells into two populations showing differing GFP-VHL localization patterns. While in 76% of *ΔΔ btn2Δ* cells GFP-VHL fluorescence remained diffuse, 21% of cells showed large, multiple (>3) GFP-VHL foci in the cytosol and no diffuse fluorescence, indicating protein aggregation and proteostasis collapse (Fig. 2a, b). Similar results were obtained when analysing *ΔΔ hsp42Δ* and *ΔΔ hsp42Δbtn2Δ* cells (Fig. 2a, b).

We also analysed whether aggregation of GFP-VHL can be observed upon loss of Hsp42 sequestrase function in *sse1Δ hsp104Δ* mutant cells, which leads to a temperature-sensitive growth phenotype at 35–37 °C (Supplementary Fig. 1b). Shifting *sse1Δ hsp104Δ hsp42Δ* cells to 37 °C caused uncontrolled aggregation of GFP-VHL, whereas *sse1Δ hsp104Δ* control cells showed diffuse fluorescence or CytoQ formation (Supplementary Fig. 3h, i). We infer that loss of sequestrase function in these cells ultimately results in uncontrolled and harmful protein aggregation. This supports a critical role of Btn2 and Hsp42 in maintaining cellular proteostasis in cells with limited Hsp70 capacity.

### Btn2 and Hsp42 maintain basal levels of Hsp70 capacity

To directly monitor the status of Hsp70 activity as a foldase in the diverse mutant strains, we made use of Luciferase-DM (R188Q/R261Q), a hyper-thermolabile variant of the Hsp70 substrate firefly Luciferase^3,53^. We genetically fused Luciferase-DM with GFP allowing to correlate Luciferase activity with subcellular localization and sequestration. Since the nuclear compartment is particularly vulnerable towards loss of sequestrase (Btn2) activity (Figs. 1a, 2a), we targeted the reporter to the nucleus by inserting the SV40 NLS, generating GFP-Luciferase-DM-NLS. At 25 °C Luciferase activities and levels were largely comparable for wt and all chaperone mutants tested (Fig. 2c). Upon continuous incubation at 28 and 30 °C, Luciferase activities remained stable in wt cells while, respectively, decreasing to 72% and 29% in *ΔΔ* cells, indicating deficiencies in Hsp70-dependent folding of GFP-Luciferase-DM-NLS (Fig. 2c). The drop of Luciferase activity at 30 °C in *ΔΔ* cells correlated with reduced levels as well as nuclear foci formation (INQ) of GFP-Luciferase-DM-NLS (Fig. 2d–f). This indicates that lowering Hsp70 capacity triages nuclear Luciferase towards both degradation and controlled sequestration pathways.

The temperature-dependent inactivation of Luciferase was further increased in *ΔΔ hsp42Δ* and *ΔΔ btn2Δ* cells, which displayed only 10% and 8% reporter activity at 30 °C, respectively (Fig. 2c). This further loss of activity was not caused by enhanced degradation as reporter levels were comparable to *ΔΔ* cells (Fig. 2d). However, instead of chaperone-dependent sequestration into nuclear deposits (INQ), we observed sequestrase-independent aggregation of GFP-Luciferase-DM-NLS as 86% of *ΔΔ btn2Δ* and 55% of *ΔΔ hsp42Δ* cells exhibited multiple, cytosolic GFP-Luciferase-DM-NLS foci (Fig. 2e, f). Diffuse nuclear reporter staining, still detectable in *ΔΔ* cells, was hardly observed in the triple mutants, explaining the extensive loss of Luciferase activity in, for example, the *ΔΔ btn2Δ* mutant cells. When comparing the time-resolved changes in GFP-Luciferase-DM-NLS localization in *ΔΔ* and *ΔΔ btn2Δ* cells upon temperature upshift from 25 to 30 °C, we observed that controlled, Btn2-dependent INQ formation in *ΔΔ* cells preceded the formation of multiple foci in *ΔΔ btn2Δ* cells (Fig. 2g). We infer that loss of the Btn2-dependent GFP-Luciferase-DM-NLS sequestration immediately after shift to non-permissive conditions causes proteostasis breakdown and uncontrolled reporter aggregation, leading to lower levels of native protein.

### Executors and sites of sequestration are interchangeable

**Fig. 3:**
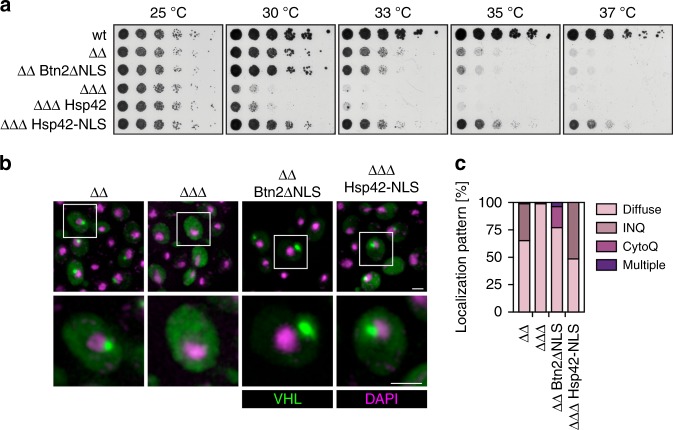
Sequestrase function per se is crucial for growth of *hsp104Δ fes1Δ* cells. **a** Serial dilutions of indicated *S. cerevisiae* wild-type (wt) and chaperone mutant cells (*ΔΔ*: *hsp104Δ fes1Δ*; *ΔΔΔ*: *hsp104Δ fes1Δ btn2Δ*) expressing Btn2ΔNLS or Hsp42-NLS were spotted on YPD plates and incubated at indicated temperatures for 2 days. **b**, **c** *Saccharomyces cerevisiae* chaperone mutant cells expressing GFP-VHL or mCherry-VHL were grown at 25 °C. DNA was stained by DAPI. Cellular localizations of VHL were determined (*n* = 100; 2 replicates). INQ and CytoQ foci were quantified depending on their position relative to the DAPI signal (INQ: directly adjacent; CytoQ: separated) by visual inspection

GFP-VHL is predominantly deposited in the nucleus of *ΔΔ* cells (Fig. 2a, b). Accordingly, *ΔΔ btn2Δ* cells are more temperature sensitive for growth as compared to *ΔΔ hsp42Δ* cells (Fig. 1a), suggesting a particular role of Btn2-driven INQ formation in cellular protection. We questioned whether the site of sequestration (nucleus vs. cytosol) and the implicated sequestration factor (Btn2 vs. Hsp42) are crucial for viability of *ΔΔ* cells. We therefore analysed whether *ΔΔ btn2Δ* cells can be rescued by (i) targeting the cytosolic Hsp42 to the nucleus (Hsp42-NLS) or (ii) directing misfolded proteins to cytosolic deposition sites by expressing Btn2-ΔNLS lacking its NLS as sole Btn2 copy (Fig. 3a, Supplementary Fig. 4a). Consequences on protein sequestration upon expression of Hsp42-NLS and Btn2-ΔNLS were monitored by use of the GFP-VHL reporter (Fig. 3b, c).

The presence of the *hsp42-NLS*, but not the *hsp42* allele, fully complemented growth deficiencies of *ΔΔ btn2Δ* cells, which even grew better than *ΔΔ* cells (Fig. 3a). Growth rescue correlated with the ability of Hsp42-NLS to efficiently restore nuclear deposition of GFP-VHL (Fig. 3b, c). To demonstrate that it is the sequestrase activity of Hsp42 that specifically rescues growth, we (i) expressed *ΔPrLD-hsp42-NLS*, lacking the PrLD, which is essential for Hsp42 sequestrase activity^18^ in *ΔΔ btn2Δ* cells or (ii) targeted Hsp26 to the nucleus of the mutant cells (Supplementary Fig. 4). ΔPrLD-Hsp42-NLS did neither restore growth nor INQ formation (Supplementary Fig. 4b, c). Similarly, expressing *hsp26-NLS* as additional copy did not rescue growth and did not induce INQ formation, documenting specificity for Hsp42 sequestrase function (Supplementary Fig. 4b, d). We cannot exclude that the C-terminal NLS fusion alters Hsp26 oligomerization and functionality, although it does not affect Hsp42 (Fig. 3). Hsp26 is overproduced in *sse1Δ hsp104Δ hsp42Δ* cells, yet cannot compensate for the lack of the cytosolic sequestrase Hsp42 (Supplementary Fig. 4e). This further underlines that Hsp42, but not Hsp26, functions as sequestrase to protect cells under conditions of low Hsp70 capacity.

Growth of *ΔΔ btn2-ΔNLS* cells was indistinguishable from *ΔΔ* cells, but exhibited cytosolic instead of nuclear GFP-VHL inclusions (Fig. 3). Therefore, sequestrase function of Btn2-ΔNLS in the cytosol is sufficient to rescue growth. We infer misfolded protein sequestration as such is most important to maintain growth of *ΔΔ* cells; the particular sequestrase (Btn2 vs. Hsp42) and sequestration sites (nucleus vs. cytosol) for misfolded proteins are in principle interchangeable. Notably, Hsp42-NLS but not Hsp42 triggers misfolded protein sequestration in *ΔΔ btn2Δ* cells. This might be explained by a higher local concentration due to the restricted space in the nucleus.

### Btn2 is a stand-alone sequestrase with sHsp-like features

**Fig. 4:**
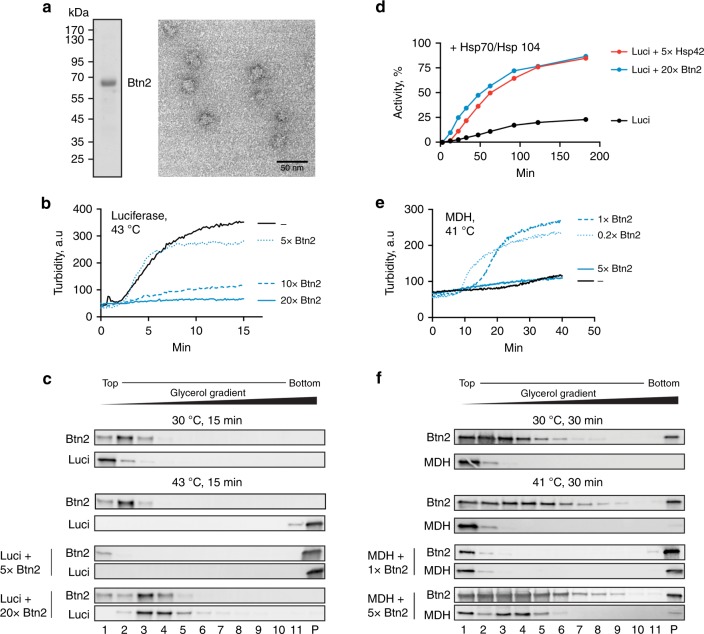
Btn2 has stand-alone chaperone and sequestrase activity. **a** The structure of purified Btn2 was analysed by negative stain electron microscopy. Scale bar: 50 nm. The purity of Btn2 (3 μg) was documented by SDS-PAGE. Btn2 runs aberrantly, which might be caused by its highly charged C-terminal domain. **b**, **c** One hundred nanomolar Luciferase was incubated in the absence and presence of Btn2 at various ratios (0.5–2 μM) at 43 °C and Luciferase aggregation was monitored by determining sample turbidity. A representative light scattering experiment is shown. Samples were additionally analysed by 10–50% glycerol gradient centrifugation and SDS-PAGE followed by western blot analysis. **d** Luciferase was aggregated at 43 °C for 15 min either alone or in the presence of a 5-fold and 20-fold excess of Hsp42 and Btn2, respectively. Luciferase activities were determined upon shifting the samples to 30 °C in the presence of the yeast bi-chaperone disaggregation system Hsp70/Hsp104 (Ssa1/Sis1/Fes1/Hsp104) and an ATP regenerating system. The activity of native Luciferase was set as 100%. A representative refolding experiment is shown. **e**, **f** Malate dehydrogenase (MDH) (0.5 μM) was denatured at 41 °C in the absence or presence of Btn2 at various ratios (0.1–2.5 μM. As control 2.5 μM Btn2 was heated alone. Protein aggregation was monitored by determining sample turbidity and a representative light scattering experiment is shown. “-“ (black line) refers to MDH-only sample. Samples were additionally analysed by glycerol gradient centrifugation as described above

Btn2 represents an uncharacterized protein, which precludes further functional comparison with Hsp42. Moreover, the notable impact of Hsp42 on Btn2-driven sequestration of misfolded proteins in the nucleus (Fig. 2a, e) raises the question whether Btn2 alone can function as sequestrase. We therefore determined the biochemical properties and chaperone functions of purified Btn2 and compared them with those of Hsp42. We first analysed the oligomeric state of Btn2 by size-exclusion chromatography (Supplementary Fig. 5a). Btn2 eluted in the void volume indicating it forms high-molecular-weight complexes reminiscent of sHsp oligomers. Negative staining electron microscopy (EM) revealed that Btn2 forms globular structures with a diameter of 15–20 nm (Fig. 4a).

We next analysed the effects of Btn2 on aggregation of thermolabile Luciferase at 43 °C in vitro. Btn2 exhibits ATP-independent holdase activity and prevented the formation of large, turbid luciferase aggregates if present in 10-fold molar excess over substrate (Fig. 4b). This is reminiscent of sHsp chaperone activity including Hsp42, which prevented Luciferase aggregation if present in 5-fold molar excess. Btn2 association with misfolded Luciferase was confirmed by glycerol gradient centrifugation and negative staining EM showing the formation of smaller Btn2–Luciferase complexes (Fig. 4c, Supplementary Fig. 5b).

When shifting Btn2–Luciferase complexes, generated at 43 °C, back to 30°C no spontaneous refolding of Luciferase was observed. This suggests that Btn2 stably sequesters Luciferase and does not release the substrate, similar to stable substrate binding by sHsps^54^. The stable nature of the sHsp–substrate complexes demands for Hsp70-Hsp100 disaggregase activity for substrate release and refolding^54–57^. Conversely, the binding of substrates to sHsps enhances refolding by disaggregases as compared to substrates aggregated in the absence of sHsps^56,58,59^. To assess whether Btn2 has similar features, we compared the reactivation of Luciferase from aggregated and Btn2-bound states by the yeast Hsp70 (Sis1/Ssa1/Fes1)–Hsp100 (Hsp104) bi-chaperone system. As a reference, we included Hsp42/Luciferase complexes, which allowed for 87% refolding of Luciferase within 180 min by the bi-chaperone system, while only 23% of Luciferase aggregated without Hsp42 was reactivated (Fig. 4d). Reactivation of Luciferase from soluble Btn2/Luciferase complexes by the Hsp70-Hsp100 bi-chaperone system (80% after 180 min) was comparable to that of Hsp42/Luciferase complexes, indicating that substrate sequestration by Btn2 also increases refolding yields (Fig. 4d). Notably, Btn2 also allowed for partial, Hsp104-independent refolding of Luciferase by the Hsp70 system alone (Supplementary Fig. 5c), again revealing a chaperone activity similar to that of sHsps^54^. We conclude that Btn2 exhibits sHsp-like chaperone activity and associates with misfolded proteins to increase their reactivation by Hsp70-Hsp100 chaperones.

Btn2 is required for the formation of nuclear sequestrations in yeast cells^13^, while preventing protein aggregation in vitro (Fig. 4b, c). This seeming discrepancy either points to additional cellular factors required for nuclear sequestration of misfolded proteins or experimental limitations of the in vitro chaperone assays employed. We recently established an assay using malate dehydrogenase (MDH) as thermolabile substrate, which allowed to document sequestrase activity of Hsp42 in vitro^45^. Here, MDH is incubated at mild denaturing temperature (41 °C) well below its melting temperature (50.9 °C). This experimental parameter mimics more closely the moderate heat stress regimes imposed onto cells when sequestrase activity is observed^11–13^. Incubation at 41 °C caused slow MDH unfolding without leading to the formation of turbid MDH aggregates (Fig. 4e, f, Supplementary Fig. 4d) in agreement with former findings^45^. The presence of sub-stoichiometric (1:0.2) and stoichiometric (1:1) MDH:Btn2 molar ratios triggered MDH “aggregation” and formation of turbid, insoluble MDH/Btn2 complexes (Fig. 4e, f). Btn2 did not accelerate MDH inactivation, excluding the possibility that Btn2 stimulates MDH “aggregation” by promoting MDH unfolding (Supplementary Fig. 5d). The addition of a high 5-fold molar excess of Btn2 led to the formation of non-turbid MDH/Btn2 complexes that were still larger as compared to MDH denatured in Btn2 absence (Fig. 4f). These findings indicate that the ratio between Btn2 and substrate is a key parameter to control the size of Btn2/substrate complexes, similar to what we observed for Hsp42^45^. Btn2 became an integral part of the complexes, demonstrating that Btn2 sequestrase activity involved direct physical interactions with misfolded MDH (Fig. 4f).

We infer that Btn2 exhibits functional similarities with Hsp42. Btn2 is necessary and sufficient to trigger sequestration of a denatured model substrate. Additional cellular factors are therefore not required to allow Btn2 to sequester proteins in vivo.

### Dissection of Btn2 domains crucial for sequestrase function

**Fig. 5:**
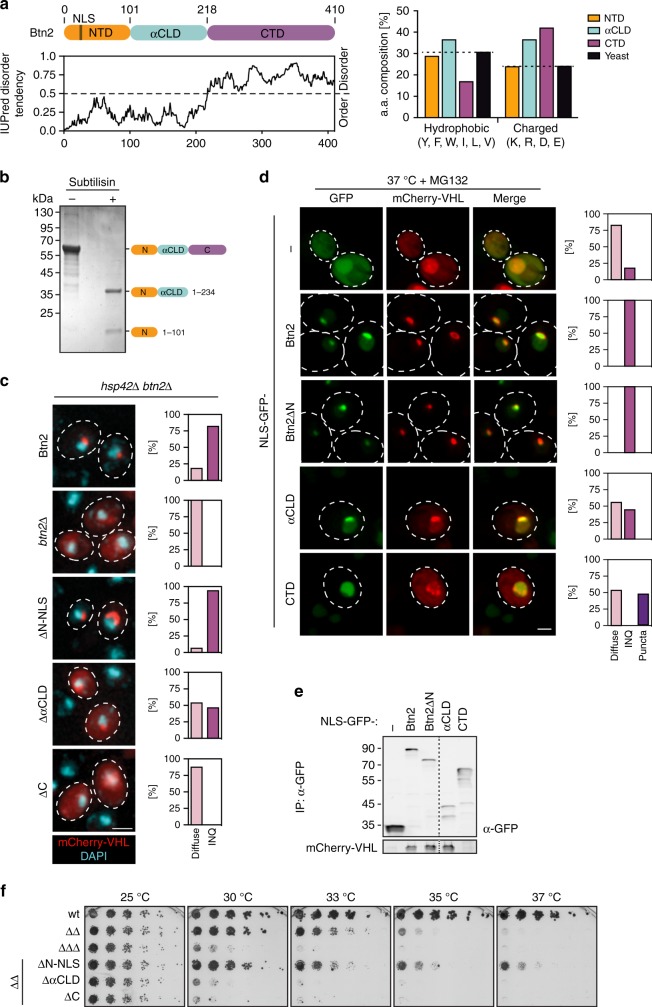
Characterization of Btn2 domains. **a** Btn2 consists of an N-terminal domain (NTD) harbouring a nuclear localization sequence (NLS), a α-crystallin-like domain (αCLD) and a disordered C-terminal domain (CTD). Protein disorder was predicted by IUPred and the relative abundance of hydrophobic and charged amino acids within each Btn2 domain was determined (right panel). The average abundance of respective amino acids in the yeast proteome is included as reference. **b** Partial proteolysis of Btn2 by subtilisin was analysed by SDS-PAGE. Btn2 degradation products were identified by mass spectrometry. **c** *Saccharomyces cerevisiae hsp42Δ btn2Δ* expressing the indicated Btn2 variants and mCherry-VHL as aggregation reporter were grown at 30 °C and heat shocked to 37 °C in the presence of MG132 for 90 min. Cellular localizations of mCherry-VHL were determined (*n* = 100, 2 replicates). DNA was stained by DAPI. **d** *Saccharomyces cerevisiae hsp42Δ btn2Δ* cells expressing mCherry-VHL and NLS-GFP or indicated NLS-GFP-Btn2 fusion constructs were grown at 30 °C and proteotoxic stress (37 °C + MG132) was applied for 90 min. Cellular localizations were determined and quantified (*n* = 100 cells, 2 replicates). INQ: single GFP and mCherry-VHL foci; puncta: multiple mCherry-VHL foci that do not co-localize with GFP. **e** *Saccharomyces cerevisiae hsp42Δ btn2Δ* cells expressing mCherry-VHL and NLS-GFP or indicated NLS-GFP-Btn2 fusion constructs were exposed to proteotoxic stress (37 °C + MG132). GFP constructs were isolated using GFP-binder beads. The amount of precipitated GFP constructs and co-isolated mCherry-VHL was determined by western blot analysis. **f** Serial dilutions of indicated *S. cerevisiae* wild-type (wt) and chaperone mutant cells (*ΔΔ* *hsp104Δ fes1Δ* *ΔΔΔ* *hsp104Δ fes1Δ btn2Δ*) expressing the indicated Btn2 deletion variants were spotted on YPD plates and incubated at indicated temperatures for 2 days

Individual Btn2 domains have not been assigned and structural data are not available, hampering mechanistic analysis of Btn2-mediated protein aggregation. To define domains of Btn2, we first performed structure prediction by hidden Markov model (HMM)–HMM comparison (HHpred)^60^. HHpred revealed that, as best hit in the PDB (Protein Data Bank) database, the central segment of Btn2 comprising residues 101–218 shows a weak predicted secondary structure similarity to the conserved α-crystallin domain of sHsps (Supplementary Fig. 6a). In similarity to α-crystallin domains, this segment is predicted to consist of seven β-sheets, which led us to refer to it as α-crystallin-like domain (αCLD). The identification of the central αCLD defines additional N-terminal (NTD, 1–100) and C-terminal (CTD, 218–410) domains for which, however, no related structure could be predicted (Fig. 5a). The NTD harbours the monopartite NLS (39KRRK42) of Btn2. Prediction of disordered and folded protein segments using IUPred^61^ suggests that the NTD and αCLD form folded structures, whereas the CTD is intrinsically disordered. This can be explained by low abundance of hydrophobic/aromatic amino acids (16.8%) and high content of charged residues (41.9%) in the CTD (Fig. 5a). Limited proteolysis of purified Btn2 by Subtilisin, which non-specifically cuts exposed protein segments, confirmed the predicted Btn2 domain organization. Subtilisin treatment generated two N-terminal Btn2 fragments comprising NTD (1–100) and NTD-αCLD (1–234) while causing rapid degradation of the CTD (Fig. 5b).

To identify domains critical for Btn2 sequestrase function, we created Btn2 deletion variants (Btn2-ΔNTD, Btn2-ΔαCLD, Btn2-ΔCTD) and C terminally fused the strong SV40 NLS to all constructs. Btn2-wt or deletion variants were expressed from the endogenous chromosomal locus in *hsp42Δ* cells co-expressing the mCherry-VHL reporter. This facilitated the functional readout as mCherry-VHL is only deposited at nuclear INQ sites in these cells (Fig. 5c)^11,13^. All constructs additionally harboured a C-terminal FLAG-tag for quantification in western blots. We found comparable production of Btn2 constructs after heat shock except Btn2-ΔCTD, which accumulated to lower levels (Supplementary Fig. 5c). mCherry-VHL accumulated to comparable levels in all *btn*2 mutant constructs tested (Supplementary Fig. 6b). Upon proteotoxic stress (37 °C + MG132), mCherry-VHL stayed diffuse in *hsp42Δ btn2Δ* cells, while Btn2-FLAG expression led to the formation of mCherry-VHL INQ foci (Fig. 5c, Supplementary Fig. 6c). Btn2-ΔNTD-NLS also efficiently triggered INQ formation, demonstrating that the NTD is dispensable for sequestrase function. In contrast deletions of αCLD and CTD affected Btn2 sequestrase activity (Fig. 5c). mCherry-VHL stayed diffuse in cells expressing Btn2-ΔCTD and also did not form INQ in the majority (54%) of cells expressing Btn2-ΔαCLD. The remaining 46% of cells showed nuclear mCherry-VHL foci, but diffuse mCherry-VHL fluorescence was still observed in these cells, indicating inefficient INQ formation (Fig. 5c).

To further define roles of Btn2 αCLD and CTD in INQ formation, we performed a complementary approach and constitutively expressed the individual domains fused to GFP in yeast *hsp42Δ btn2Δ* cells. GFP fusions were tested for their ability to sequester mCherry-VHL at INQ. All GFP fusions harboured an additional N-terminal NLS ensuring nuclear localization and were produced at levels similar or higher as compared to GFP-Btn2 (Supplementary Fig. 6d). mCherry-VHL formed nuclear foci already at 30 °C in cells expressing GFP-Btn2, but not in cells expressing GFP fusions to single αCLD and CTD domains (Supplementary Fig. 6e). mCherry-VHL deposition at INQ at 30 °C is explained by constitutive expression of GFP-Btn2, whereas endogenous Btn2 only accumulates upon heat shock (e.g. to 37 °C). In NLS-GFP expressing control cells, mCherry-VHL was diffusely distributed at 30 °C and proteotoxic stress triggered its nuclear accumulation in a diffused state, indicating preferential nuclear import under stress conditions consistent with former observations^13,62,63^. While GFP-αCLD and GFP-CTD did not allow for INQ formation of mCherry-VHL at 30 °C, some degree of nuclear mCherry-VHL foci formation became apparent after stress application (Fig. 5d). The partial stress-dependent mCherry-VHL sequestration mediated by GFP-αCLD or GFP-CTD can be likely explained by nuclear enrichment of mCherry-VHL upon heat shock. This will increase the local concentration of mCherry-VHL and thereby facilitate sequestration. The sequestrase activity of the GFP fusions to isolated αCLD and CTD, however, was minor as compared to full-length Btn2, as judged by the fact that diffuse nuclear staining of mCherry-VHL was still present, in agreement with the phenotypes of respective deletion constructs.

The pattern of stress-induced mCherry-VHL foci formed in the presence of GFP-αCLD and GFP-CTD differed. GFP-CTD expression caused formation of multiple mCherry-VHL foci at the nuclear periphery, which, however, did not co-localize with GFP-CTD. While this finding points to a role of Btn2-CTD in INQ formation, in agreement with INQ deficiency of Btn2-ΔCTD-expressing cells, it does not allow to define a precise role. GFP-αCLD expression led to the formation of a single nuclear mCherry-VHL focus, which always co-localized with GFP-αCLD, suggesting sequestration of the reporter through direct GFP-αCLD interaction. We probed for mCherry-VHL interaction by isolating GFP-Btn2 fusion constructs after stress using GFP binder and testing for co-purified mCherry-VHL (Fig. 5e). mCherry-VHL was co-isolated with GFP-Btn2 and GFP-αCLD, but not GFP-CTD or the NLS-GFP control, demonstrating binding of αCLD to the misfolded substrate.

We finally tested the ability of the Btn2 deletion constructs to restore growth of *ΔΔ btn2Δ* cells at elevated temperatures (Fig. 5f). Btn2-ΔαCLD and Btn2-ΔCTD did not rescue the growth deficiency of *ΔΔ btn2Δ* cells, mirroring their defects in INQ formation. Surprisingly, Btn2-ΔNTD-NLS did not only complement the growth defects of *ΔΔ btn2Δ* cells, but even allowed for partial growth at 37 °C, which was not observed for *ΔΔ* reference cells expressing Btn2-wt (Fig. 5d). Btn2-ΔNTD-NLS therefore exhibits a gain-of-activity phenotype and is superior to Btn2-wt if expressed in *ΔΔ* cells with low Hsp70 capacity. We assume that the underlying molecular basis is independent of sequestrase activities as (i) Btn2-wt and Btn2-ΔNTD-NLS do not differ in INQ formation (Fig. 5c) and (ii) both were expressed at comparable levels (Supplementary Fig. 6f). We instead speculate that differences in the susceptibility of INQ towards protein disaggregases might explain the noticed differences between Btn2-wt and Btn2-ΔNTD-NLS in complementation behaviour.

### Btn2-NTD recruits disaggregases for INQ solubilization

**Fig. 6:**
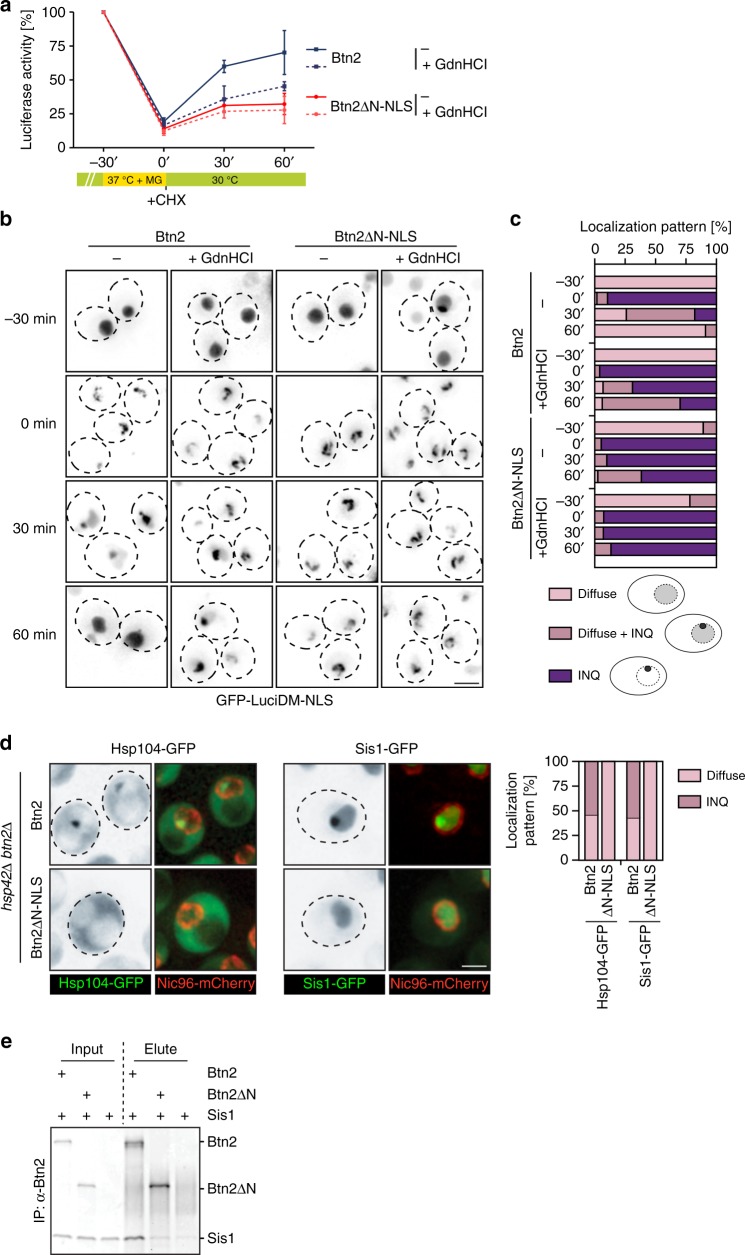
The Btn2-NTD recruits disaggregases for INQ solubilization. **a**–**c** *Saccharomyces cerevisiae hsp42Δ* cells expressing GFP-LuciDM-NLS and Btn2 or Btn2ΔN-NLS were grown at 30 °C and exposed to proteotoxic stress for 30 min (37 °C + MG132). Cycloheximide (CHX) was added upon shifting cells back to 30 °C. Luciferase activities and cellular localizations were determined and quantified (*n* = 100) at indicated time points. Luciferase activities prior to stress application were set to 100% and standard deviations were calculated based on three replicates. The Hsp104 disaggregase was inhibited by growing cells in presence of 3 mM guanidinium hydrochloride (GdnHCl). GFP-LuciDM-NLS localization were determined at the indicated time points (**b**) and quantified (**c**) for *n* = 100 cells and 2 replicates. **d** *Saccharomyces cerevisiae hsp42Δ* cells expressing Btn2 or Btn2ΔN-NLS and either Hsp104-GFP or Sis1-GFP were grown at 30 °C and exposed to proteotoxic stress (37 °C + MG132). Cellular localizations of Hsp104-GFP and Sis1-GFP were determined (*n* = 100, 2 replicates). The nuclear envelope was labelled by coexpression of fluorescent Nic96-mCherry nucleoporin. **e** Sis1 was incubated with Btn2 or Btn2ΔN and Btn2 was isolated by the addition of Btn2-binder beads. Input and bounds fractions were analysed by SDS-PAGE followed by SYPRO-Ruby staining

The above results prompted us to investigate whether Btn2-mediated sequestrations are reversed during recovery from stress, and if so, what the contribution of the Btn2 domains, especially of the NTD, to this process might be. We expressed Luciferase-DM-GFP-NLS as aggregation reporter in Btn2-wt- and Btn2-ΔNTD-NLS-expressing cells and subjected them to proteotoxic stress (37 °C + MG132) for 30 min This caused strong reduction of Luciferase activity and sequestration into INQ, to similar extents for Btn2-wt- and Btn2-ΔNTD-NLS-expressing cells (Fig. 6a–c). To monitor the recovery of sequestered, pre-existing Luciferase-DM-GFP-NLS, we added cycloheximide at the end of the stress treatment to block de novo synthesis. The stress-inactivated Luciferase-DM-GFP-NLS regained 60% of its activity in Btn2-wt cells during a 60 min recovery period at 30 °C; this recovery was two-fold reduced for cells expressing Btn2-ΔNTD-NLS (Fig. 6a). To determine the contributions of the misfolded soluble and sequestered populations to Luciferase refolding, we repeated the recovery experiments in presence of low guanidinium hydrochloride (GdnHCl) concentrations (3 mM), which specifically inhibits the Hsp104 disaggregase^64^. The presence of GdnHCl reduced Luciferase refolding only in Btn2-wt-expressing cells but not in Btn2-ΔNTD-NLS-expressing cells, resulting in similar refolding yields for Btn2-wt- and Btn2-ΔNTD-NLS-expressing cells (Fig. 6a). This indicates that the increased Luciferase refolding yield in Btn2-wt cells stems from Hsp104-dependent INQ disaggregation. The partial regain of reporter activity in Btn2-ΔNTD-NLS-expressing cells instead stems from misfolded, yet soluble Luciferase-DM-GFP-NLS, which does not require Hsp104 activity for refolding.

This conclusion was confirmed when monitoring the fate of INQ-deposited Luciferase-DM-GFP-NLS in Btn2-wt- and Btn2-ΔNTD-expressing cells during the recovery phase (Fig. 6b, c). Nuclear Luciferase-DM-GFP-NLS sequestrations were efficiently removed at 30 °C within 60 min in Btn2-wt cells in an Hsp104-dependent manner, whereas they remained stable in Btn2-ΔNTD-NLS cells (Fig. 6b, c). This finding demonstrates that solubilization of Btn2-induced INQ requires the NTD of Btn2. Notably, nuclear aggregates of Luciferase-DM-GFP-NLS that formed infrequently in *btn2Δ* cells were removed in an Hsp104-dependent manner, indicating that the disaggregation machinery does not require Btn2 per se (Supplementary Fig. 7a, b). Together, these findings indicate that the active sequestration of Luciferase-DM-GFP-NLS by Btn2 induces a strong dependence of the disaggregases on the Btn2-NTD.

To substantiate these findings, we used mCherry-VHL as alternative sequestration reporter (Supplementary Fig. 7c). We expressed mCherry-VHL in *hsp42Δ* cells expressing Btn2-wt or Btn2-ΔNTD-NLS and induced INQ formation by proteotoxic stress (37 °C + MG132) for 90 min Btn2-wt- and Btn2-ΔNTD-NLS-induced deposition of mCherry-VHL at INQ with similar efficiencies and both co-localized with the sequestered reporter (Supplementary Fig. 7c, d). During recovery (30 °C and MG132 washout), mCherry-VHL foci were efficiently removed within 120 min in cells expressing Btn2-wt (Supplementary Fig. 7c), whereas they persisted in cells expressing Btn2-ΔNTD-NLS, confirming that the Btn2-NTD plays a crucial role in INQ disaggregation.

Btn2 accumulates only during the immediate phase of heat stress application, but is rapidly degraded afterwards^12,13^. We hypothesized that the Btn2-ΔNTD-NLS fragment is stabilized and consequently causes INQ persistence. We therefore compared the levels of Btn2 and Btn2-ΔNTD-NLS after heat shock and during the recovery period (Supplementary Fig. 7e). Btn2-wt and Btn2-ΔNTD-NLS accumulated to comparable levels upon heat shock and Btn2-wt rapidly vanished afterwards. The vast majority (71%) of Btn2-ΔNTD-NLS was also degraded upon stress relief (60 min at 30 °C), excluding the possibility that stabilization of Btn2-ΔNTD-NLS prevents INQ solubilization. However, a minor fraction (29%) of Btn2-ΔNTD-NLS remained stable throughout the recovery period (Supplementary Fig. 6E). We speculate that this fraction is protected from degradation because it is an integral part of the stabilized INQ. Accordingly, partial Btn2-ΔNTD-NLS stabilization is a consequence but not a cause of INQ persistence.

To address why the Btn2-NTD is crucial for INQ disaggregation, we tested whether it promotes direct interaction with components of the Hsp70-Hsp100 bi-chaperone disaggregase. We first monitored the localization of Hsp104-GFP in stressed *hsp42Δ* cells expressing either Btn2-wt or Btn2-ΔNTD-NLS (Fig. 6d). Hsp104-GFP formed stress-induced nuclear INQ foci in the presence of Btn2-wt but not Btn2-ΔNTD-NLS, indicating that Hsp104 targeting to INQ is abolished (Fig. 6d, Supplementary Fig. 7f). Btn2-ΔNTD-NLS efficiently induced INQ formation under identical stress conditions (Fig. 5c, d), excluding differences in INQ formation as reason for the absence of Hsp104-GFP foci.

Hsp104 is recruited to protein aggregates by cooperation with Ssa1, which in turn requires Hsp40 co-chaperones for aggregate targeting^65,66^. Sis1 is a major Hsp40 in the nucleus of yeast^62^ and cooperates with Ssa1 and Hsp104 in aggregate solubilization (Fig. 4d). We therefore tested for Sis1-GFP binding to INQ induced by either Btn2-wt or Btn2-ΔNTD-NLS (Fig. 6d, Supplementary Fig. 7f). Sis1 associated with INQ in the presence of Btn2-wt as revealed by formation of nuclear Sis1-GFP foci, but stayed diffuse in the nucleus in the presence of Btn2-ΔNTD-NLS. We infer that the yeast disaggregase (Hsp104/Ssa1/Sis1) does not bind INQ formed in the presence of Btn2-ΔNTD-NLS, explaining INQ persistence.

The determined binding deficiency of Sis1 suggests a physical interaction between Sis1 and the Btn2-NTD. Indeed, Sis1 physically interacted with Btn2-wt but not Btn2-ΔNTD *in vitro*, demonstrating that NTD mediated interaction of both proteins (Fig. 6e). Ydj1, the major yeast Hsp40 co-chaperone, did not interact with Btn2 (Supplementary Fig. 7g), excluding that Sis1 recognizes Btn2 as misfolded protein. The specific Btn2-Sis1 interaction is in agreement with former findings^12^. Yet, we place the interaction of both chaperones in a distinct cellular context by showing that it is required for recruitment of the yeast Hsp70-Hsp100 disaggregases to ensure rapid INQ solubilization during recovery phases. The Btn2-NTD-mediated recruitment of Sis1 and consequently Ssa1 can explain the superior growth complementation of *ΔΔ* cells by Btn2-ΔNTD-NLS (Fig. 5f): the Btn2 mutant does not recruit Hsp70 to INQ, thereby further increasing Hsp70 capacity and improving growth at increased temperatures in cells with low Hsp70 capacity.

## Discussion

The organized sequestration of misfolded proteins by specialized chaperones termed sequestrases is only recently realized as third pillar of protein quality control, next to refolding and degrading pathways. Here, we position this activity within the proteostasis network of yeast cells, demonstrate its crucial cytoprotective function and the direct, functional, and physical linkage of sequestrases to the Hsp70 system.

**Fig. 7:**
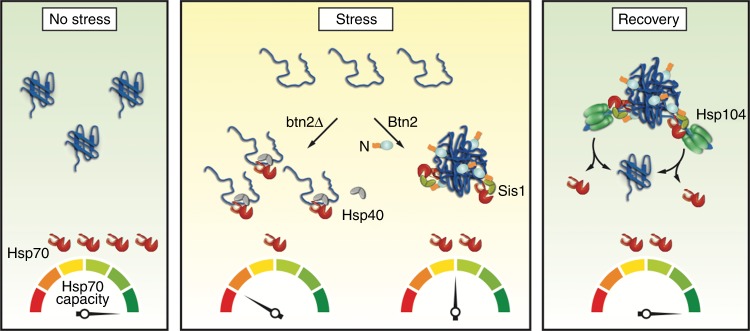
Btn2 sequestrase prevents Hsp70 overload during stress. Non-stressed cells have a high Hsp70 capacity, which is strongly reduced upon stress application by accumulating soluble misfolded proteins. Btn2 sequesters misfolded proteins into large assemblies and attenuates drop of Hsp70 capacity. The Btn2 N-terminal domain (N) recruits the cellular disaggregation machinery (Hsp70/Hsp104) by interacting with the Hsp70 co-chaperone Sis1. This ensures rapid reflux of sequestered proteins into the proteostasis network during recovery periods

We show that the sequestrases Btn2 and Hsp42 become essential for yeast growth upon genetically limiting the capacity of Hsp70. Sequestration most likely shields sticky surfaces of misfolded proteins, thereby decreasing the number of available binding sites for Hsp70. This seems to be a critical function during stress exposure of cells since it counteracts an over boarding titration of Hsp70 away from its cellular housekeeping functions such as de novo protein folding^67^. In consequence, sequestrase activity effectively prevents proteostasis collapse and ensures cell viability under stress (Fig. 7). Protein aggregation has been frequently observed in Hsp70 mutant cells^68–71^, but was viewed as disastrous consequence of proteostasis breakdown. We suggest that this event rather reflects an activated cellular emergency programme executed by the Hsp42 and Btn2 sequestrases. Btn2 and Hsp42 are strongly upregulated upon both heat shock and limitations of Hsp70 capacity (Fig. 1c)^72–74^. This further supports that sequestration is an immediate response to acute proteotoxic stress, counteracting Hsp70 depletion.

We observed a dominant role of Btn2 and nuclear sequestration in stress survival of *fes1Δ hsp104Δ* cells. Most likely, nuclear Hsp70 capacity is particularly limited in these cells as they lack the nuclear Hsp70 NEF, Fes1. Hsp70 limitations may directly impact nuclear functions such as cell cycle control and gene regulation^75^. Notably, in *sse1Δ hsp104Δ* cells, which lack the cytosolic Hsp70 NEF, Sse1, cytosolic Hsp42 becomes the most important sequestrase (Supplementary Fig. 1b). The particular roles of Btn2 and Hsp42 thus match compartment-specific limitations of the Hsp70 system.

How does Btn2 execute sequestrase activity? We present a comprehensive structure–function analysis of Btn2. We show Btn2 has chaperone activity and shares characteristics with sHsps. It exerts a stand-alone sequestrase activity necessary and sufficient to organize protein sequestration without additional factors. Our analysis defines three Btn2 domains (NTD, αCLD, CTD) with distinct functions in INQ formation and solubilization. Attempts to purify Btn2-ΔαCLD and Btn2-ΔCTD remained unsuccessful and characterizing the functions of αCLD and CTD domains therefore relied on the in vivo analysis of respective deletion constructs. The αCLD and CTD are essential for INQ formation with the αCLD involved in substrate interaction. We note that Cur1, the yeast paralog of Btn2, is largely missing the CTD and, accordingly, is not required for INQ formation^12^. The CTD is largely disordered but its precise role in sequestration remains unknown. Frequently proteins with large disordered segments are involved in organized phase separation and sequestration processes^18,76,77^ by undergoing multivalent interactions with themselves and other components. The CTD of Btn2 contains multiple small clusters of negatively and positively charged residues, which we speculate promote Btn2 self-interaction but also contacts with sequestered substrates.

The Btn2-NTD is not essential for INQ formation but required for INQ solubilization. The NTD specifically recruits the Hsp70 co-chaperone Sis1, thereby initializing a hierarchical binding cascade of Hsp70 and Hsp104, which together form the yeast disaggregase. Btn2 was previously shown to bind to the C-terminal dimerization motif of Sis1 that is non-essential for its co-chaperone function^12,78^. Btn2-Sis1 interaction therefore employs domains that are not crucial for basic sequestrase (Btn2) or disaggregase (Sis1) activities.

The function of Btn2-NTD as binding partner for Sis1 demands for its exposure at the INQ surface. Btn2 seems to shield the INQ surface, as Sis1 cannot bind to INQ induced by Btn2-ΔNTD-NLS, although it can directly bind to protein aggregates in vitro^45,79^. Sis1 dependence on Btn2-NTD for INQ binding therefore indicates that sequestered substrates are not sufficiently accessible to allow for direct Sis1 binding. This INQ architecture provides specificity for Sis1 and Hsp70-Hsp104 disaggregases. This may impact fate decisions of INQ-sequestered substrates, which get a chance to refold upon disaggregation as shown here for Luciferase-DM-GFP-NLS. Accordingly, yeast endogenous proteins that become sequestered upon heat shock in the cytosol and the nucleus are disaggregated and refolded without degradation occurring during the recovery period^5^. The ability of Btn2 to provide Hsp70 selectivity, involving physical contacts with the Hsp40 co-chaperone, Sis1, is a rather unique feature since the cooperation between Hsp40/Hsp70 and classical sHsps does not require direct interactions^55^.

In summary, we show that the sequestrase Btn2 controls key aspects of nuclear protein sequestration. By sequestering misfolded proteins during acute stress periods, Btn2 prevents Hsp70 overload (Fig. 7). At the same time, it prepares the sequestered proteins for fast and efficient Hsp70-dependent recovery upon stress relief.

## Methods

### Yeast strains, plasmids and growth conditions

All *S. cerevisiae* strains used in this study are derived from BY4741. Strains and used plasmids are listed in the Supplementary Table 1, and used primers are listed in Supplementary Table 2. Yeast gene deletions were performed using a PCR-based protocol^80^. Deletion cassettes harbouring different selection markers were constructed by PCR using specific primers containing homologous flanks (30–50 nucleotides) for the gene of interest. PCR products were transformed into yeast cells, which were subsequently plated on appropriate plates containing the antibiotics or lacking the corresponding amino acid for selection. Gene deletions were confirmed by PCR and western blot analysis.

Antibiotics were used at the following final concentrations: 300 μg/ml (geneticin), 100 μg/ml (nourseothricin) and 300 μg/ml (hygromycin). Doxycycline was added in a final concentration of 10 μg/ml. Yeast cultures were cultivated in liquid YPD media or SC media supplemented with 2% glucose or 2% galactose/raffinose. The corresponding solid media contained 2% (w/v) agar. Yeast cultures were grown at 25 °C or at otherwise indicated growth conditions.

### Growth assays

For spot test yeast cells were grown in SC or YPD medium to log phase (optical density at wavelength 600 nm (OD_600_) = 0.6–0.8) and then diluted to OD_600_ = 0.2. Cells were 5-fold serially diluted and spotted on YPD or SC agar plates. Plates were incubated at diverse temperatures for 2 days before documentation.

For recording growth curves yeast cells were grown in SC or YPD medium to log phase. Cells were diluted to OD_600_ = 0.2 and aliquoted into a 24-well plate. Yeast growth curves were measured in a temperature-controlled microplate reader (SPECTROstar Nano, BMG Labtech).

### Biochemical assays

Hsp104, Ssa1, Sis1, Ydj1, Fes1 and Luciferase were purified from *E. coli* BL21 (NEB) or XL1 blue (lab stock) cells^45,81^. Hsp42 was purified from *E. coli* ArticExpress cells (Agilent). All proteins were produced in cells grown at 30 °C, except for Luciferase and Hsp42, which were produced at 16 or 13 °C, respectively. Ssa1 and Fes1 were produced as His_6_-SUMO fusion constructs, and Hsp42 was produced as MBP (maltose-binding protein) fusion construct. Hsp104- and Luciferase-harboured C-terminal His_6_ tags and were purified by Protino Ni-IDA resin in LEW buffer (50 mM NaH_2_PO_4_ pH 8.0, 300 mM NaCl, 5 mM β-mercaptoethanol) and Superdex S200 size-exclusion chromatography in buffer A (50 mM HEPES, pH 7.6, 150 mM KCl, 20 mM MgCl_2_, 2 mM dithiothreitol (DTT)) supplemented with 5% (v/v) glycerol. His_6_-tagged Ydj1 and Sis1 were purified by Protino Ni-IDA resin in LEW buffer. His_6_-SUMO-Ssa1 and His_6_-SUMO-Fes1 were purified by Protino Ni-IDA resin in LWB150 buffer (40 mM HEPES, pH 7.4, 150 mM KCl, 5 mM MgCl_2_, 10 mM β-mercaptoethanol, 5% (v/v) glycerol). Eluted proteins were dialysed against LWB150 in the presence of His_6_-Ulp1 at 4 °C overnight to cleave off His_6_-SUMO. His_6_-SUMO and His_6_-Ulp1 were renived by incubation with Protino Ni-IDA resin. MBP-Hsp42 was purified by amylose resin (NEB) in Hsp42 buffer (50 mM Tris, pH 7.5, 200 mM NaCl, 2 mM DTT, 10% (v/v) glycerol). Eluted proteins were dialysed overnight in the presence of PreScission protease (Sigma) to cleave of MBP. Hsp42 was finally purified by Sephacryl S-300 size-exclusion chromatography in Hsp42 buffer. Malate dehydrognease was purchased from Roche, and pyruvate kinase was purchased from Sigma-Aldrich. Btn2 and Btn2∆N were produced as His_6_-SUMO fusion proteins in *E. coli* BL21DE3 (NEB) harbouring pCool6-Btn2 or pCool6-Btn2∆N. Cells were lysed in LEW buffer (50 mM NaH_2_PO_4_, pH 8.0, 300 mM NaCl, 1 mM DTT) and incubated with Protino Ni-IDA resin. Btn2 and Btn2∆N were eluted from the resin by His_6_-Ulp1 cleavage in LEW buffer containing 50 mM NaCl. Btn2 and Btn2∆N were further purified by anion exchange column (Resource Q, Amersham) using a linear NaCl gradient (50–550 mM NaCl in LEW buffer) and size-exclusion chromatography (Superdex 200, Amersham) in LEW buffer.

For determining the impact of Btn2 on protein aggregation, 0.1 μM Luciferase or 0.5 μM MDH were incubated alone or in the presence of Btn2 at 43 and 41 °C, respectively, in buffer B (50 mM HEPES, pH 7.6, 50 mM KCl, 5 mM MgCl_2_, 2 mM DTT). Protein aggregation was monitored by determining sample turbidity at 600 nm using a Perkin-Elmer luminescence spectrometer LS50B.

Protein aggregates or complexes with Btn2 were analysed by glycerol gradient centrifugation. Luciferase (0.1 μM) or MDH (0.5 μM) were incubated at 43 or 41 °C for 15 and 60 min, respectively, in the absence or presence of Btn2. Protein samples (100 μl) were overlaid on top of a 10–50% glycerol gradient in buffer A containing 1 μM bovine serum albumin (BSA). Samples were centrifuged at 215,000 × *g* for 1 h at 4 °C (SW60 rotor). Fractions (500 μl) were collected and analysed by sodium dodecyl sulfate-polyacrylamide gel electrophoresis (SDS-PAGE) followed by western blot analysis.

The impact of Btn2 and Hsp42 on substrate recovery was determined in disaggregation assays. Luciferase (0.1 μM) was incubated at 43 °C for 15 min in buffer A in the absence and presence of 0.5 μM Hsp42 or 2 μM Btn2. Samples were shifted to 30 °C and mixed with disaggregating chaperones (2 μM Ssa1, 1 μM Sis1, 1 μM Fes1, 1 μM Hsp104, 50 nM final concentration of Luciferase) together with an ATP regenerating system (2 mM ATP, 3 mM phosphoenolpyruvate, 20 ng/μl pyruvate kinase). Luciferase activities were determined using the Lumat LB 9507.

In limited proteolysis experiments, 8 μg Btn2 and 5 ng subtilisin (Boehringer) in 20 μl reaction in buffer B (50 mM Tris, pH 7.5, 150 mM NaCl, 5 mM MgCl_2_, 1 mM DTT) were incubated at 25 °C for 2 h. Protease-resistant fragments were analysed by SDS-PAGE followed by Coomassie staining. Fragment identities were determined by mass spectrometry after tryptic digest.

Interaction of Btn2 and Sis1 was monitored in pull-down assays. One micromolar Btn2, Btn2∆N, Sis1 or Ydj1 were incubated in buffer A containing cOmplete Protease Inhibitor (Sigma-Aldrich) at 25 °C for 15 min before mixing with 10 μl Btn2-binder beads (Btn2 antibodies conjugated to NHS-activated sepharose beads (GE Healthcare)) for 15 min. The Btn2-binder beads were washed seven times with buffer A. Bound proteins were eluted by boiling in SDS sample buffer and analysed by SDS-PAGE stained by SYPRO-Ruby (Thermo Fisher Scientific).

Western blot analysis was performed as follows: SDS-PAGEs were transferred to PVDF membranes by semi-dry blotting. Membranes were subsequently blocked with 3% BSA (w/v) in TBS-T (Tris-buffered saline with Tween-20). Antibodies and used dilutions are listed in Supplementary Table 3 Anti-rabbit alkaline phosphatase conjugate (Vector Laboratories) was used as secondary antibody (1:20,000). Blots were developed using ECF™ Substrate (GE Healthcare) as reagent and imaged via Image-Reader LAS-4000 (Fujifilm). Western blotting was performed in two or more independent experiments each and representative results are provided.

### FACS analysis

To monitor Hsf1-dependent gene expression, GFP was placed under the control of the *btn2* promoter. To increase reporter sensitivity, the unstable N-terminal domain of Btn2 was fused C- terminally to GFP. GFP fluorescence (excitation: 488 nm; emission: 530 nm) was determined by FACS (fluorescence-activated cell sorting) using the BD FACS Canto machine. Cultures of yeast strains were grown at 25 or 30 °C until log phase (0.6–0.8 OD). Two hundred microliters of cell culture was added to a flat-bottom 96-well plate with eight technical replicates per strain. FACS measured GFP fluorescence of 10,000 cells per strain. The GFP values were normalized by subtracting the values of a negative control.

### Negative staining and electron microscopy

Protein samples were adsorbed to glow-discharged carbon-coated EM grids for 1 min. Grids were washed with distilled water and then stained with freshly prepared 2% uranyl-formiate (two passages). Micrographs were taken at pixel sizes of 0.36 nm using a Zeiss EM912 electron microscope (Carl Zeiss, Oberkochen, Germany) equipped with a cooled CCD camera (Proscan, München, Germany).

### Immunoprecipitation

Fifty milliliters of yeast culture was grown at 30 °C to mid-log-phase and cells were harvested. Cell pellets were resuspended in 500 μl IP buffer (50 mM Tris, pH 7.5, 150 mM NaCl, 5 mM MgCl_2_, 0.1% (v/v) TX-100, 5% (v/v) glycerol, 1 mM DTT, 2 mM phenylmethanesulfonyl fluoride, cOmplete Protease Inhibitor) and lysed by bead beater (FastPrep-24, speed 6.5, 30 s, 6 repeats). After removing the cell debris, 2 mg of total protein was incubated with 50 μl GFP-binder beads in 1 ml IP buffer at 4 °C for 1 h. The beads were washed 7× with IP buffer and bound proteins were eluted by boiling in SDS sample buffer. The amount of precipitated GFP and co-precipitated mCherry-VHL were monitored by western blotting using specific antibodies.

### Microscopy and image processing

For live cell imaging, yeast cells were harvested by centrifugation, resuspended in SC medium and directly applied onto microscope slides. For DAPI (4′,6-diamidino-2-phenylindole) staining, cells were first fixed with cold 70% ethanol for 5 min, washed once with H_2_O, followed by incubation with 50 ng/ml DAPI in phosphate-buffered saline (PBS). To collect cell images, optical sections of 0.2 µm were acquired to image the whole-cell volume using a widefield system (xCellence IX81, Olympus) equipped with a Plan-Apochromat ×100/NA 1.45 oil immersion objective and an EMCCD camera (Hamamtsu). Acquired z-stacks were deconvolved with xCellence software (Olympus) using the Wiener filter. All further processing and analysis of digital images was performed with ImageJ (NIH).

### Immunofluorescence microscopy

Immunostaining was performed as previously described^13^. In brief, yeast cells were fixed with 4% (v/v) *p*-formaldehyde/100 mM KPO_4_ (Sigma-Aldrich) for 1 h followed by cell wall digestion by 500 µg/ml Zymolase T-100 in wash buffer (1.2 M sorbitol/100 mM KPO_4_, pH 6.5) supplemented with 20 mM β-mercaptoethanol for 30 min at 30 °C. Spheroblasts were attached to poly-lysine-coated cover slides, permeabilized by washing three times with 1% (v/v) Triton X-100/100 mM KPO_4_, pH 6.5 and blocked for 1 h with 1% (w/v) BSA in 100 mM KPO_4_, pH 6.5. All antibodies were diluted in blocking buffer. Primary and secondary antibody incubations were carried out for 1 h at room temperature. Afterwards, spheroblasts were stained with 100 ng/ml DAPI in PBS and embedded in 50% glycerol. Antibodies and used dilutions are listed in Supplementary Table 2.

## Supplementary information

Supplementary Information

Source Data

## Data Availability

The source data underlying Figs. 1c, 2c, d, 4b–f, 5e, 6a, e, Supplementary Figs. 1c, e, 2a–c, e, 3b, d, e, 4a, e, 5c, d, 6b, d, f, 7e, g are provided as a Source data file. Other data are available from the corresponding authors upon reasonable request.
