# Supplementary material for: Cellular sequestrases maintain basal Hsp70 capacity ensuring balanced proteostasis
Source: Nat Commun. 2019 Oct 24;10:4851. doi: 10.1038/s41467-019-12868-1 (PMC6813348; doi:10.1038/s41467-019-12868-1)
Supplement: Supplementary file 1 — Supplementary Information [file 41467_2019_12868_MOESM1_ESM.pdf]

Suppl Figure 1

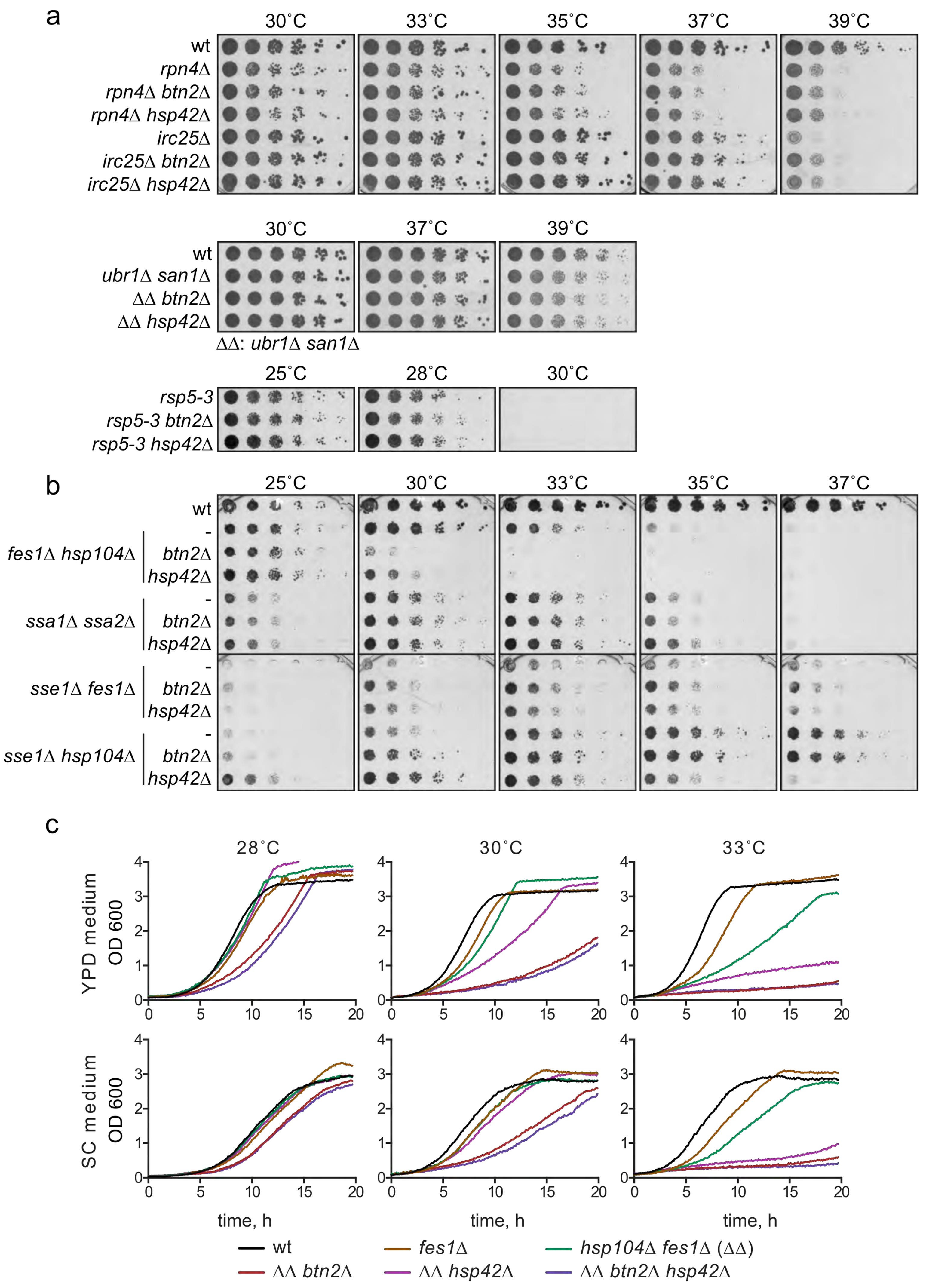

Suppl Figure 1

d

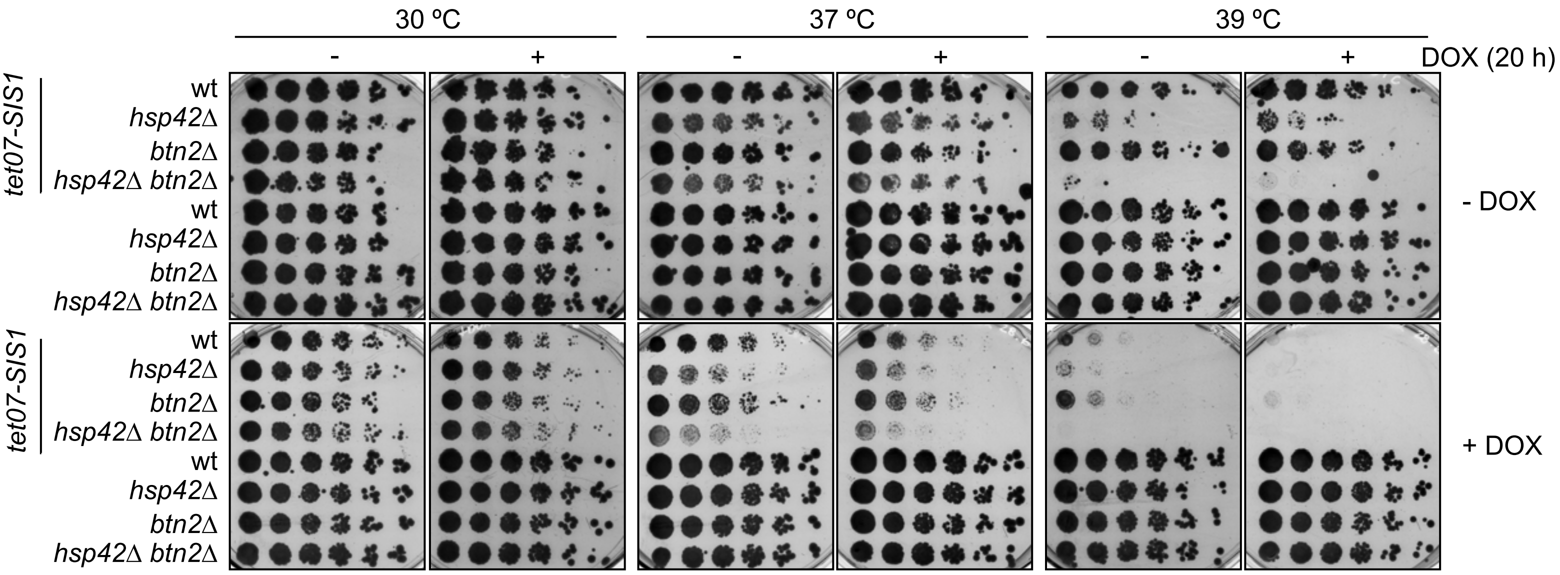

e

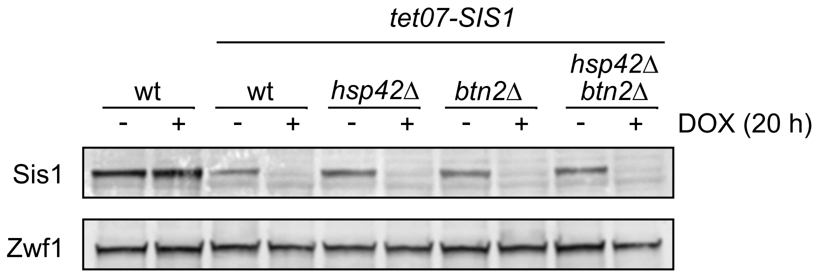

Suppl Figure 2

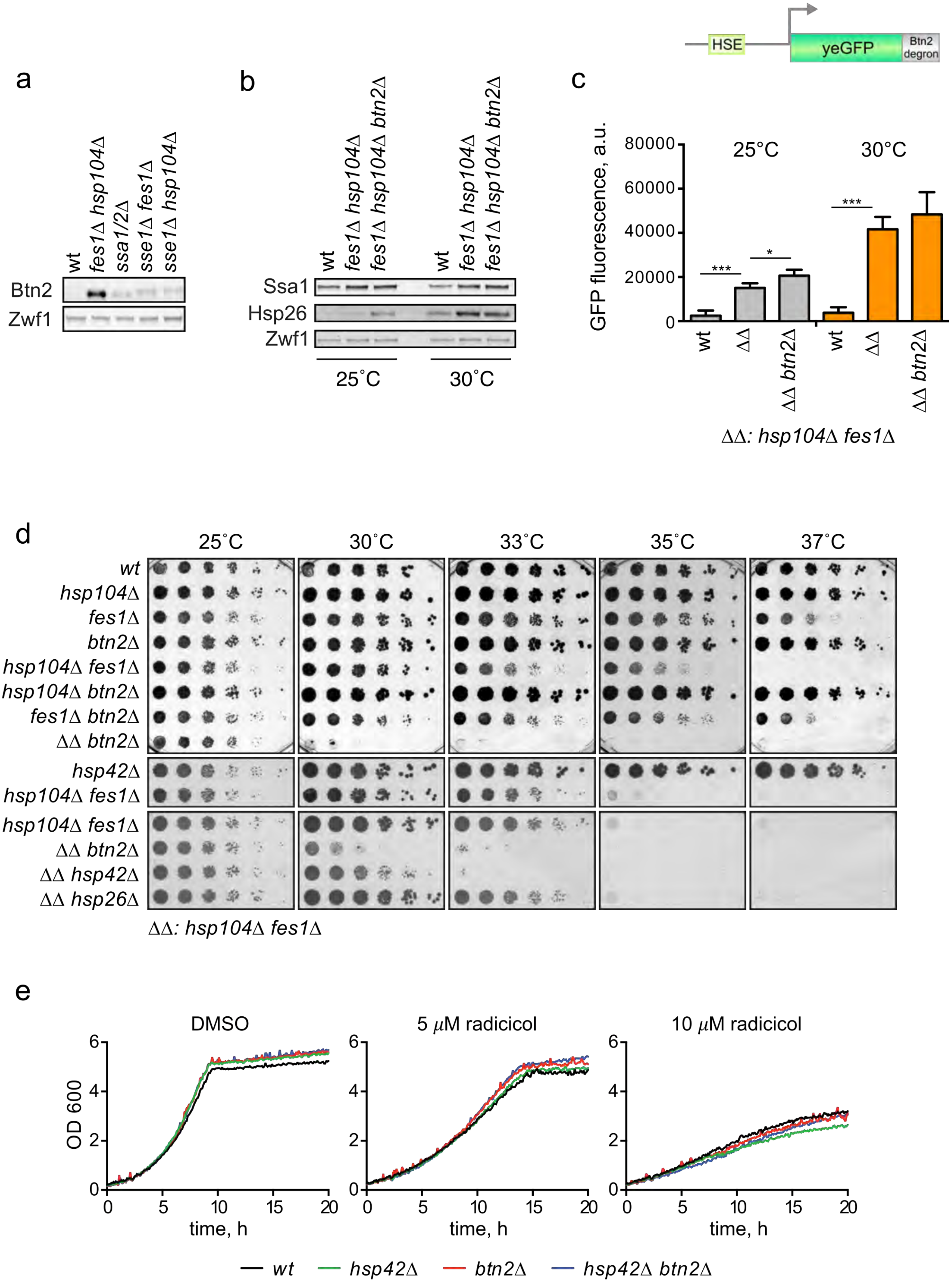

Suppl Figure 3

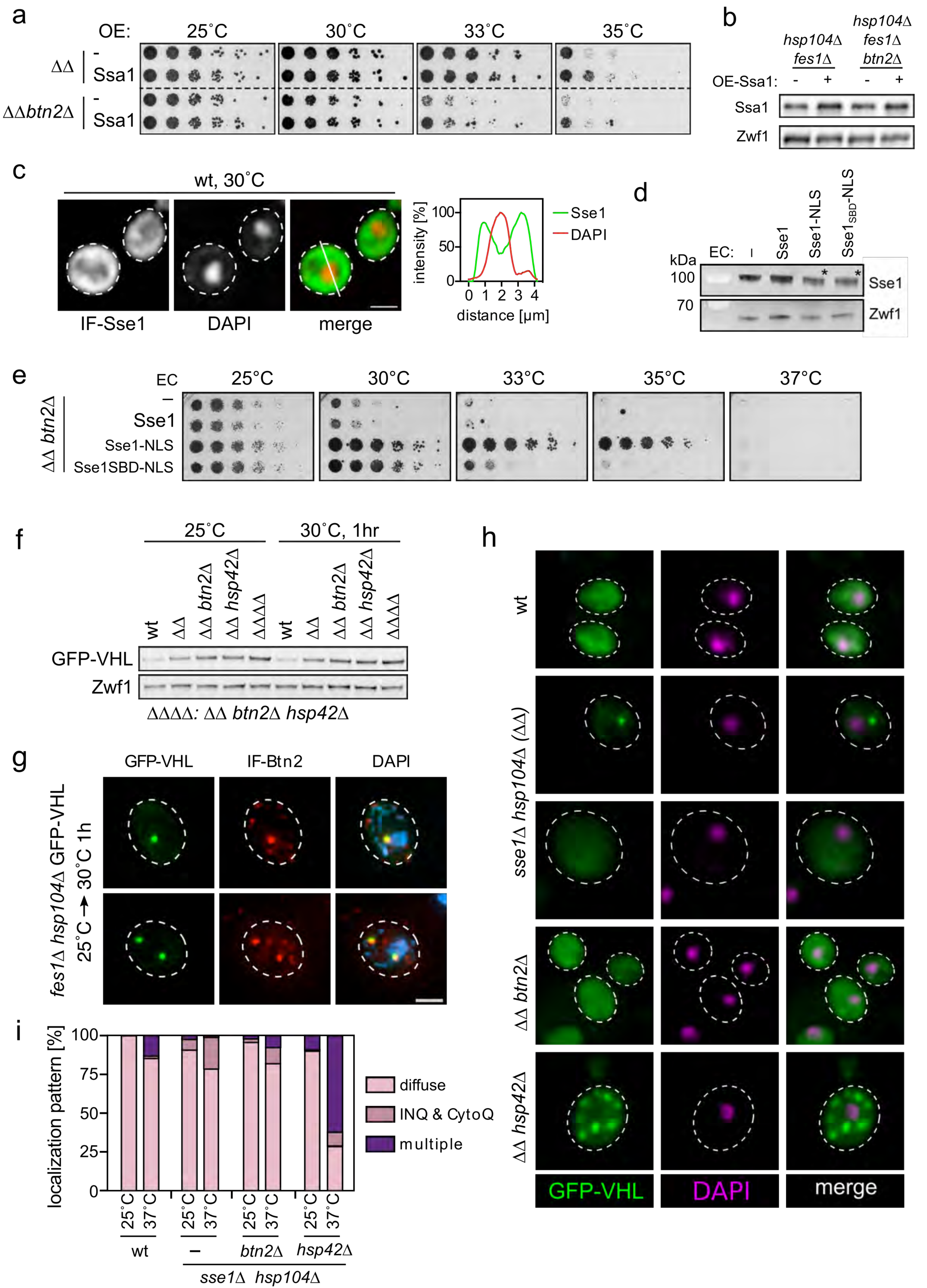

Suppl Figure 4

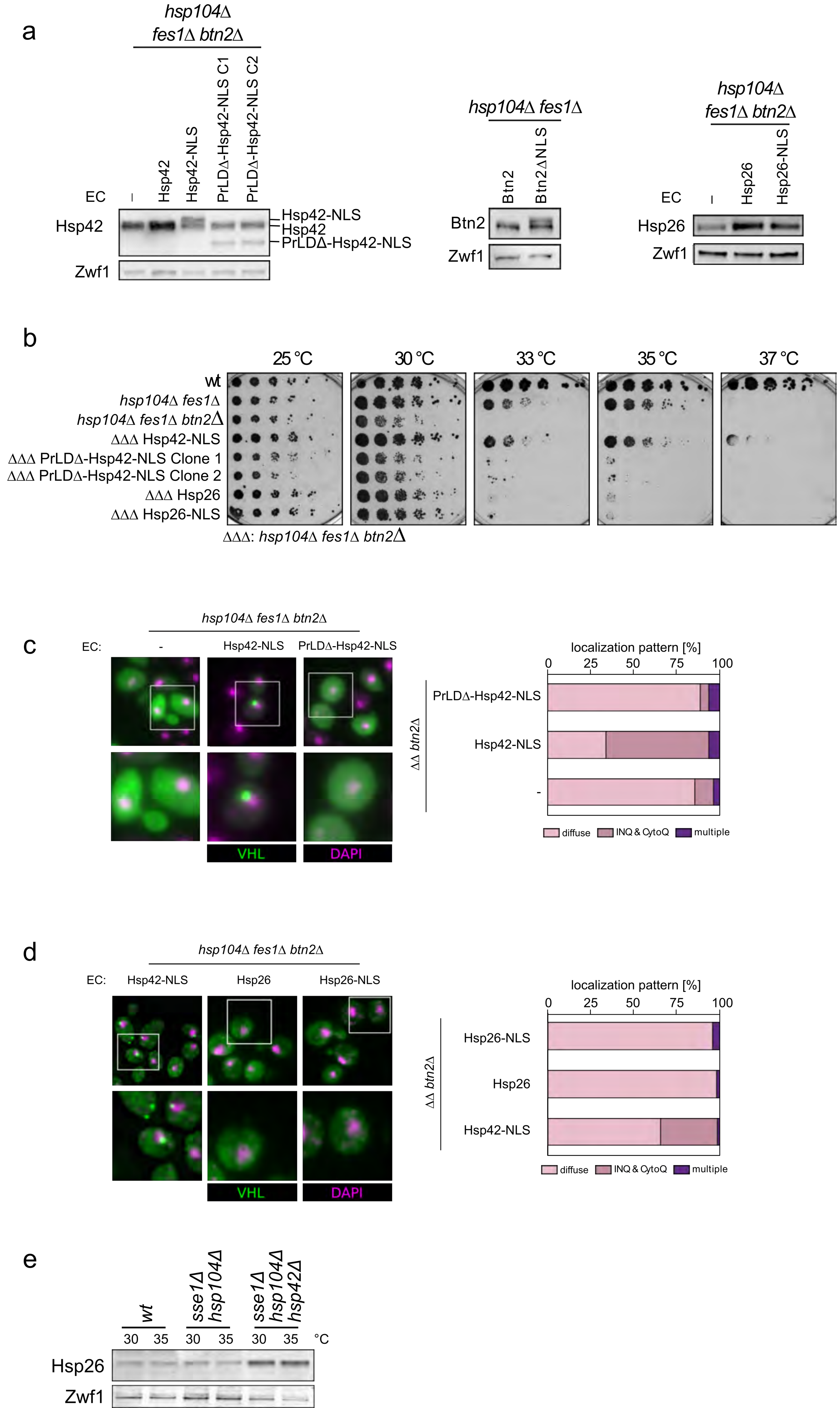

Suppl Figure 5

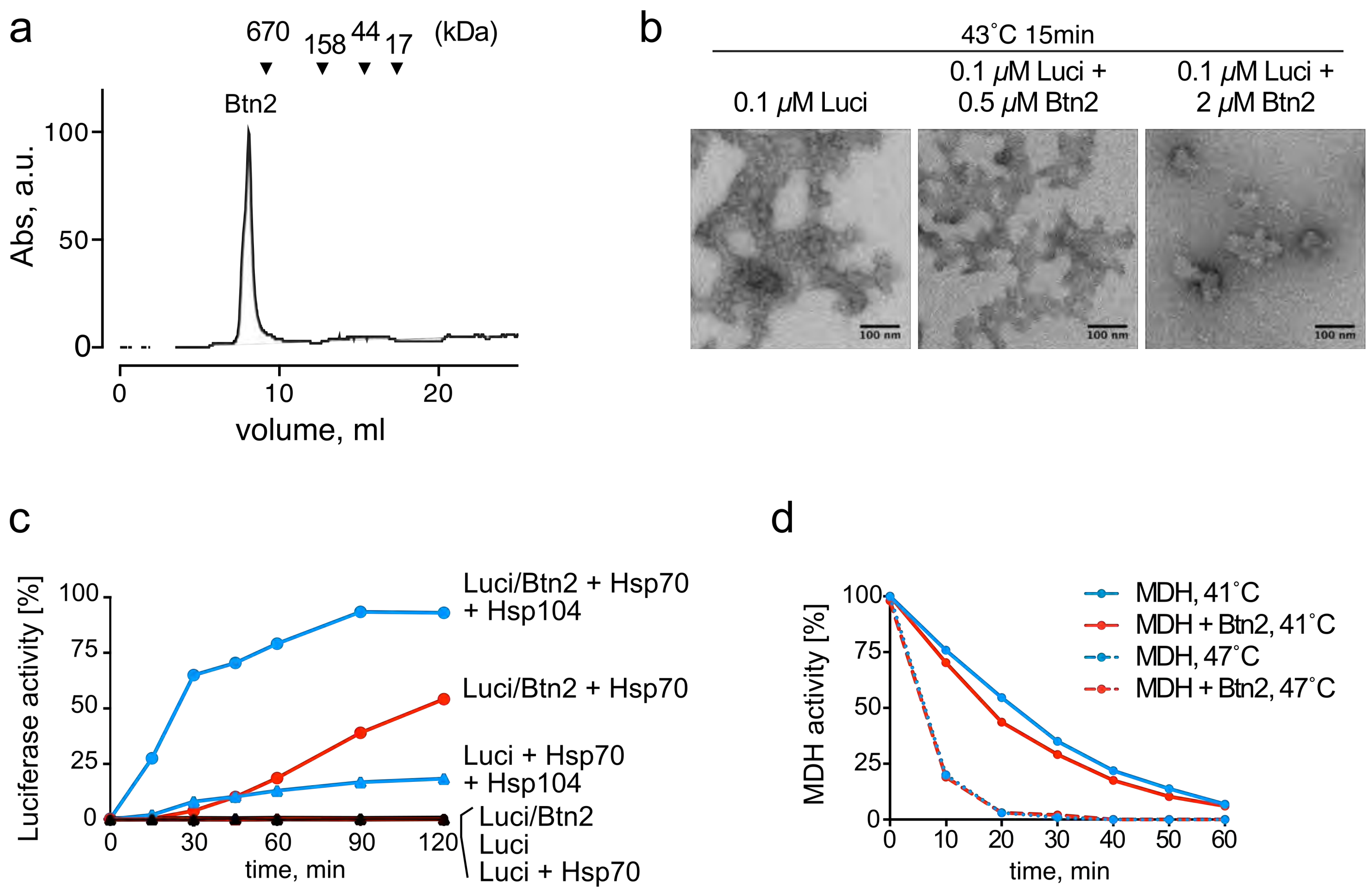

Suppl Figure 6

--

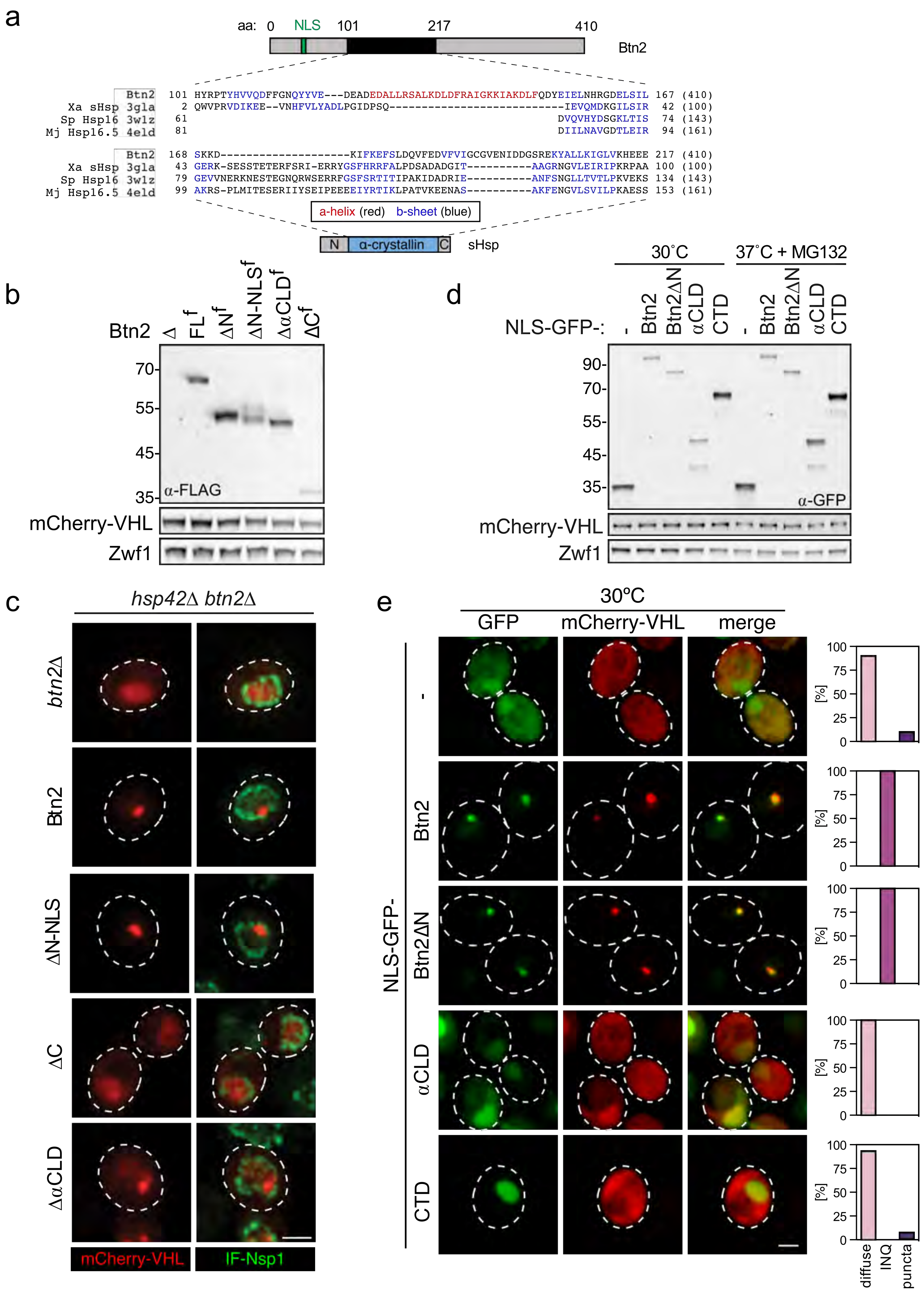

Suppl Figure 6

f

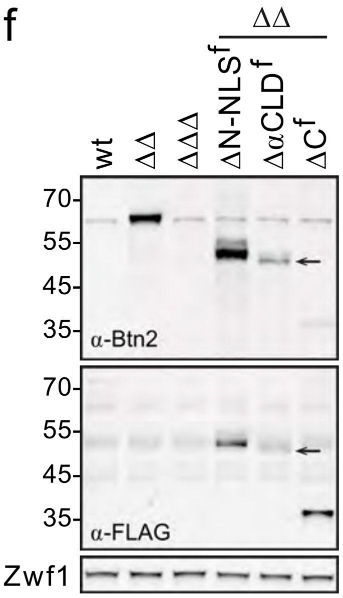

Suppl Figure 7

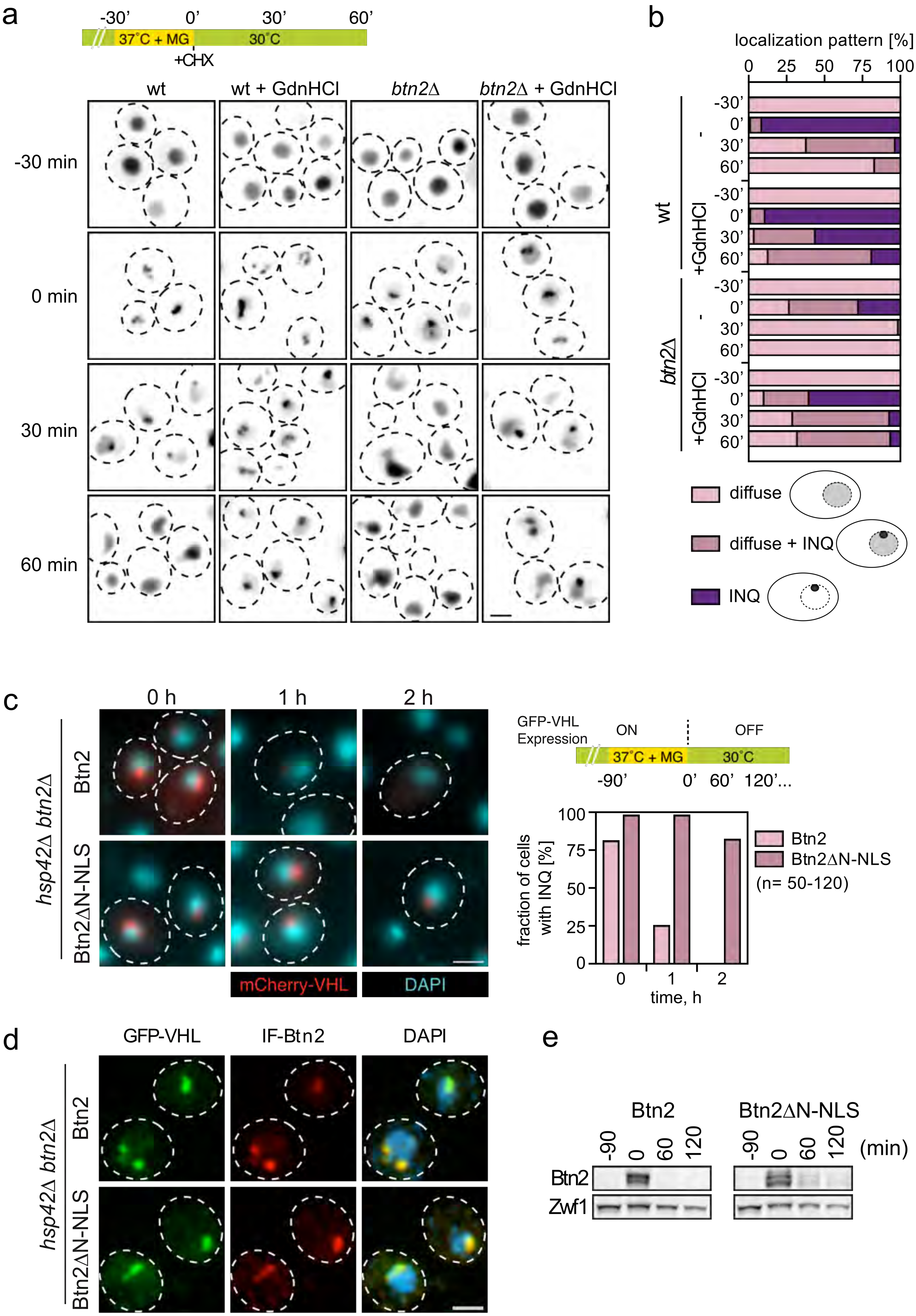

Suppl Figure 7

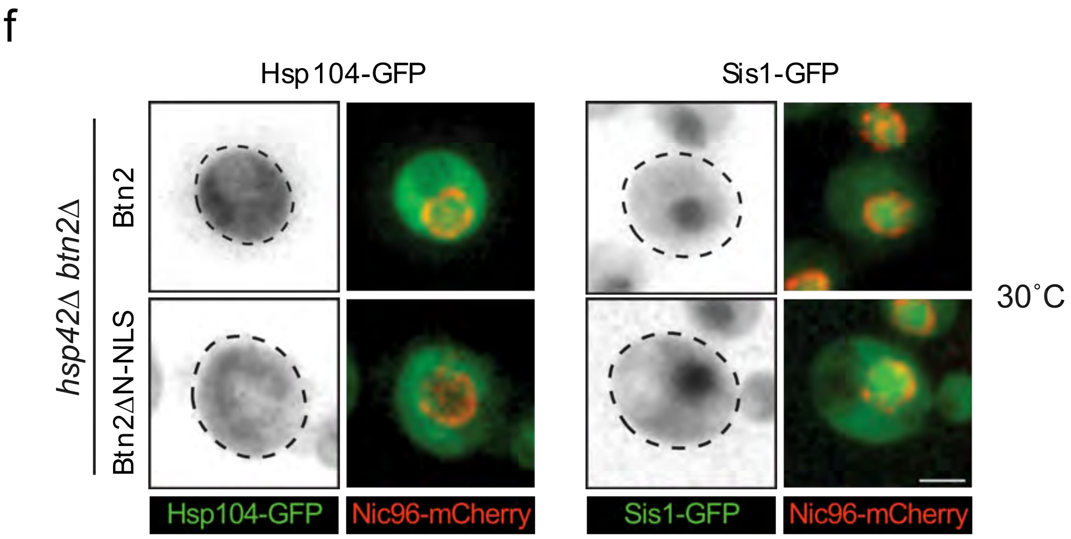

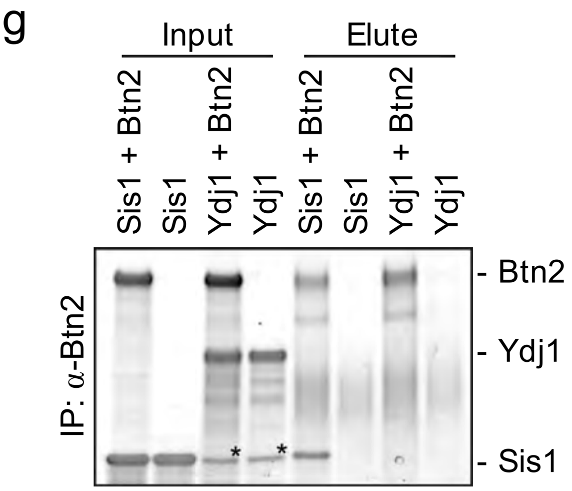

Supplementary Figure legends

Supplementary Figure 1

Probing for genetic interactions of *S. cerevisiae* sequestrases. (a/b) Serial dilutions of *S. cerevisiae* wild type (wt) and indicated mutant cells (*ΔΔ: ubr1Δ san1Δ*) were spotted on YPD-plates and incubated at indicated temperatures for 2 days. (c) Growth curves of *S. cerevisiae* wild type (wt) and indicated chaperone mutant cells (*ΔΔ: hsp104Δ fes1Δ*) were recorded at 28°C, 30°C and 33°C in either YPD or SC media. (d) *S. cerevisiae* wild type (wt) and indicated mutant cells were grown in absence or presence of doxycycline (DOX) for 20 h in YPD media. The endogenous promoter of Sis1 was replaced by doxycycline-repressible *tet07-sis1*. Cells were spotted on YPD-plates including or not including DOX and incubated at the indicated temperatures for 2 days. (e) *S. cerevisiae* wild type (wt) and indicated mutant cells were grown in absence or presence of doxycycline (DOX) for 20 h in YPD media. Sis1 levels were determined by western blot analysis and Zwf1 levels are provided as loading control.

Supplementary Figure 2

Probing for genetic interactions of *S. cerevisiae* sequestrases. (a) Btn2 levels were determined in *S. cerevisiae* wt and indicated chaperone mutant cells grown at 30°C by western-blot analysis. Zwf1 levels were determined as loading control. (b) Ssa1 and Hsp26 levels were determined in *S. cerevisiae* wt and indicated chaperone mutant cells grown at 25°C or 30°C by western-blot analysis. Zwf1

levels were determined as loading control. (c) *S. cerevisiae* wt and indicated chaperone mutant cells expressing GFP from an Hsf1-controlled promoter (P_{HSE}) were grown at 25°C or 30°C. GFP levels were determined by FACS analysis. Standard deviations were calculated based on three independent experiments. The N-terminal domain of Btn2 was fused to the GFP reporter, rendering the reporter unstable and highly sensitive. Significance was determined by t-test (***: $p < 0.01$; *: $p = 0.016$). (d) Serial dilutions of indicated *S. cerevisiae* wild type (wt) and chaperone mutant cells (*ΔΔ: hsp104Δ fes1Δ*) were spotted on YPD-plates and incubated at indicated temperatures for 2 days. (e) Growth curves of *S. cerevisiae* wild type (wt) and indicated chaperone mutant cells were recorded in YPD medium at 37°C in absence and presence of the Hsp90 inhibitor radicicol (5-10 μM).

Supplementary Figure 3

Protective function of organized protein aggregation. (a/b) Serial dilutions of *S. cerevisiae hsp104Δ fes1Δ* (*ΔΔ*) and *hsp104Δ fes1Δ btn2Δ* (*ΔΔΔ*) cells without and with Ssa1 overexpression (OE) were spotted on SC-plates and incubated at indicated temperatures for 2 days. Ssa1 levels were determined by western blot analysis and Zwf1 levels are provided as loading control. (c) The cellular localization of Sse1 was determined in *S. cerevisiae* wild type (wt) cells by immunofluorescence. DNA was stained by DAPI and a line intensity profile corresponding to the indicated line was calculated by using Image J. (d) Sse1 levels of *hsp104Δ fes1Δ btn2Δ* (*ΔΔΔ*) harbouring an extra copy (EC) of either Sse1, Sse1-NLS or $Sse1_{SBD}$-NLS were determined by western blot analysis. Zwf1 levels

are provided as loading control. “*” indicates NLS-fused Sse1. (e) Serial dilutions of *ΔΔ btn2Δ* cells expressing an additional copy of either Sse1, Sse1-NLS or $Sse1_{SBD}$-NLS were spotted on YPD plates and incubated at indicated temperatures for 2 d. (f) *S. cerevisiae* wild type (wt) and chaperone mutant cells (*ΔΔ: hsp104Δ fes1Δ; ΔΔΔΔ: hsp104Δ fes1Δ btn2Δ hsp42Δ*) were grown at 25°C and shifted to 30°C for 1 h. Levels of GFP-VHL were determined and Zwf1 levels are provided as loading control. (g) *S. cerevisiae hsp104Δ fes1Δ* cells expressing GFP-VHL were grown at 25°C and shifted to 30°C. Cellular localizations of GFP-VHL and Btn2 were determined. DNA was stained by DAPI. GFP-VHL and Btn2 foci are located adjacent to (INQ) or apart from (CytoQ) nuclear DNA. (h/i) *S. cerevisiae* wild type (wt) and indicated chaperone mutant cells expressing GFP-VHL were grown at 25°C and shifted to 37°C for 1 h. Cellular localizations of GFP-VHL were determined and the fraction of cells showing CytoQ or multiple (n >3) cytosolic foci were determined (n=100, 2 replicates). DNA was stained by DAPI.

Supplementary Figure 4

Sequestrase activity of nuclear Hsp42 is specifically required to rescue *hsp104Δ fes1Δ btn2Δ* cells. (a) Western blot analysis of yeast strains. Left: *S. cerevisiae hsp104Δ fes1Δ btn2Δ* cells harbouring an extra copy (EC) of either Hsp42, Hsp42-NLS or PrLDΔ-Hsp42-NLS were grown at 25°C and Hsp42 levels were determined by western blot analysis. Zwf1 levels are provided as loading control. Middle: *S. cerevisiae hsp104Δ fes1Δ* expressing Btn2 wild type or Btn2ΔNLS from the endogenous locus were grown at 25°C and Btn2 levels were determined by

western blot analysis. Zwf1 levels are provided as loading control. Right: *S. cerevisiae hsp104Δ fes1Δ btn2Δ* cells harbouring an extra copy (EC) of either Hsp26 or Hsp26-NLS were grown at 25°C and Hsp26 levels were determined by western blot analysis. Zwf1 levels are provided as loading control. (b) Serial dilutions of indicated *S. cerevisiae* mutant strains were spotted on YPD-plates and incubated at indicated temperatures for 2 days. (c/d) Indicated *S. cerevisiae* chaperone mutant cells expressing GFP-VHL were grown at 25°C. DNA was stained by DAPI. Cellular localizations of VHL were determined (n=100; 2 replicates). INQ and CytoQ foci were quantified depending on their position relative to the DAPI signal (INQ: directly adjacent; CytoQ: separated) by visual inspection. The varying degrees of INQ deposition of GFP-VHL (c/d) can be explained by the use of different plasmids harbouring either galactose-inducible GFP-VHL (c: pESC-GFP-VH) or constitutively expressed GFP-VHL (d: pRS303-GPD-GFP-VHL). (e) Hsp26 levels were determined in *S. cerevisiae* wt and indicated chaperone mutant cells grown at 30°C or 35°C by western-blot analysis. Zwf1 levels were determined as loading control.

Supplementary Figure 5

Biochemical characterization of Btn2. (a) Oligomerisation of Btn2 (10 μM) was analyzed by Superdex S200 HR10-30 size exclusion chromatography. The positions of a protein standard are given. (b) Negative stain electron microscopy pictures of Luciferase (Luci) which was aggregated alone or in presence of a 5- and 20-fold excess of Btn2. Scale bar 100 nm. (c) 0.1 μM Luciferase was aggregated at 43°C for 15 min either alone or in presence of a 20-fold excess of

Btn2. Luciferase activities were determined upon shifting the samples to 30°C in presence of the yeast Hsp70 chaperone system (Ssa1/Sis1/Fes1) and an ATP regenerating system. The activity of native Luciferase was set as 100%. A representative refolding experiment is shown. (d) 0.5 μM Malate Dehydrogenase (MDH) was incubated at 41°C or 47°C in absence or presence of 0.5 μM Btn2 and loss of MDH activity was determined. The activity of the native enzyme was set as 100%. A representative unfolding experiment is shown.

Supplementary Figure 6

Structure-function analysis of Btn2. (a) An internal segment of Btn2 shows structural homology to the α-crystallin domain of sHsps. Btn2 structure prediction was performed using HHpred (https://toolkit.tuebingen.mpg.de/#/tools/hhpred). A structure-based sequence alignment of Btn2 to indicated sHsps is provided; protein data bank codes are given. Predicted (Btn2) and determined (sHsp) secondary structure elements are highlighted. (b/c) *S. cerevisiae hsp42Δ btn2Δ* cells expressing mCherry-VHL and the indicated Btn2 deletion variants from the endogenous locus were grown at 30°C and shifted to 37°C in presence of MG132 for 90 min. All Btn2 variants harboured a C-terminal FLAG tag to determine Btn2 expression levels by western blot analysis using anti-Flag antibodies (α-Flag). Additionally mCherry-VHL and Zwf1 levels were determined (b). Cellular localizations of mCherry-VHL after stress treatment. The nuclear envelope was stained by Nsp1 immunofluorescence (c). (d/e) *S. cerevisiae hsp42Δ btn2Δ* expressing NLS-GFP or

indicated NLS-GFP-Btn2 fusion constructs were grown at 30°C and shifted to 37°C in presence of MG132 for 90 min. Levels of NLS-GFP constructs were determined by western blot analysis using GFP-specific antibodies (α-GFP). Additionally mCherry-VHL and Zwf1 levels were determined (d). Cellular localizations of mCherry-VHL and NLS-GFP constructs at 30°C prior to stress treatment. mCherry-VHL patterns were quantified (n=100) (e). (f) Levels of Btn2 and indicated deletion variants were determined in *S. cerevisiae hsp104Δ fes1Δ* (*ΔΔ*) cells by western blot analysis using Btn2 and Flag-specific antibodies (α-Btn2, α-Flag). Btn2 levels in wild type (wt) and *hsp104Δ fes1Δ btn2Δ* (*ΔΔΔ*) are provided as reference and control. Btn2 deletion constructs harboured a C-terminal Flag-tag to monitor expression.

Supplementary Figure 7

The Btn2 NTD is crucial for INQ solubilisation by the Hsp70-Hsp104 disaggregase machinery (a/b). *S. cerevisiae* wild type (wt) and *btn2Δ* cells expressing GFP-LuciDM-NLS were grown at 30°C in absence or presence of 3 mM guanidinium hydrochloride (GdnHCl). Cells were exposed to proteotoxic stress (37°C + MG132) for 30 min and cycloheximide (CHX) was added immediately before cellular recovery. For recovery cells were washed to remove MG132 and incubated at 30°C. Cellular localizations of GFP-LuciDM-NLS were determined and quantified (n=100). (c) *S. cerevisiae hsp42Δ* cells expressing Btn2 or Btn2ΔN-NLS from the endogenous locus were grown at 30°C. Cells additionally expressed mCherry-VHL from a galactose inducible promoter. Cells were exposed to

proteotoxic stress (37°C + MG132) for 90 min (0 h). Next, MG132 and galactose were removed and cells were incubated at 30°C for 2 h in presence of glucose to shut-off mCherry-VHL expression. Cellular localizations of mCherry-VHL were determined at the indicated time points and quantified (n>50). DNA was stained with DAPI. mCherry-VHL foci formation adjacent to nuclear DNA documented presence of INQ. (d) *S. cerevisiae hsp42Δ* cells expressing Btn2 or Btn2ΔN-NLS from the endogenous locus and GFP-VHL were grown at 30°C and proteotoxic stress (37°C + MG132) was applied for 90 min. Cellular localization of GFP-VHL and Btn2 (Immunofluorescence: IF) were determined. DNA was stained by DAPI. (e). *S. cerevisiae hsp42Δ* cells expressing Btn2 or Btn2ΔN-NLS from the endogenous locus were grown at 30°C and exposed to proteotoxic stress (37°C + MG132) for 90 min. Next MG132 was removed and cells were incubated for 2 h at 30°C. Levels of Btn2 and Btn2ΔN-NLS were determined at indicated time points by western blot analysis. (f) *S. cerevisiae hsp42Δ* cells expressing Btn2 or Btn2ΔN-NLS from the endogenous locus and either Hsp104-GFP or Sis1-GFP were grown at 30°C. Cellular localizations of Hsp104-GFP and Sis1-GFP were determined. The nuclear envelope was labelled by coexpression of fluorescent Nic96-mCherry nucleoporin. (g) Sis1 or Ydj1 was incubated with Btn2 and Btn2 was isolated by addition of Btn2-binder beads. Input and bounds fractions were analyzed by SDS-PAGE followed by SYPRO-Ruby staining.

Supplementary Table 1

S. cerevisiae strains and plasmids used in the study

Yeast strains

name	feature	genotype	Source
BY4741	wild type	BY4741, MATa; his3-Δ1; leu2-Δ0; met15-Δ0; ura3-Δ0	EUROSCARF
SMY94	hsp104Δ	hsp104Δ::loxP_URA3_loxP	this study
CHY347	fes1Δ	fes1Δ::his3MX6	this study
CHY335	hsp104Δ fes1Δ	hsp104Δ::loxP_URA3_loxP, fes1Δ::hphNT1	this study
CHY336	hsp104Δ fes1Δ btn2Δ	hsp104Δ::loxP_URA3_loxP, btn2Δ::natNT2, fes1Δ::hphNT1	this study
CHY338	hsp104Δ fes1Δ hsp42Δ	hsp104Δ::loxP_URA3_loxP, fes1Δ::hphNT1, hsp42Δ::kanMX	this study
CHY339	hsp104Δ fes1Δ btn2Δ hsp42Δ	hsp104Δ::loxP_URA3_loxP, btn2Δ::natNT2, fes1Δ::hphNT1, hsp42Δ::kanMX	this study
CHY334	hsp104Δ btn2Δ	hsp104Δ::loxP_URA3_loxP, btn2Δ::natNT2	this study
CHY367	hsp104Δ fes1Δ btn2Δ SSE1	hsp104Δ::loxP_URA3_loxP, fes1Δ::hphNT1, Sse1::Leu2	this study
CHY368	hsp104Δ fes1Δ btn2Δ SSE1-NLS	hsp104Δ::loxP_URA3_loxP, fes1Δ::hphNT1, Sse1-NLS::Leu2	this study
CHY436	rpn4Δ	rpn4Δ::kanMX	this study
CHY437	rpn4Δ btn2Δ	rpn4Δ::kanMX, btn2Δ::natNT2	this study
CHY438	rpn4Δ hsp42Δ	rpn4Δ::hphNT1, hsp42Δ::kanMX	this study
CHY439	irc25Δ	irc25Δ::kanMX	this study
CHY440	irc25Δ btn2Δ	irc25Δ::kanMX, btn2Δ::natNT2	this study
CHY441	irc25Δ hsp42Δ	irc25Δ::hphNT1, hsp42Δ::kanMX	this study
CHY91	ubr1Δ san1Δ	san1Δ::kanMX, ubr1Δ::hphNT1	this study
CHY170	ubr1Δ san1Δ btn2Δ	san1Δ::kanMX, ubr1Δ::hphNT1, btn2Δ::his3MX6	this study
CHY171	ubr1Δ san1Δ hsp42Δ	san1Δ::kanMX,	this study

		ubr1Δ::hphNT1, hsp42Δ::his3MX6	
rsp5-3	rsp5-3	rsp5-3::kanMX	this study
CHY449	rsp5-3 btn2Δ	rsp5-3::kan, btn2Δ::natNT2	this study
CHY450	rsp5-3 hsp42Δ	rsp5-3::kan, hsp42Δ::klURA3	this study
CHY406	ssa1Δ ssa2Δ	ssa1Δ::hphNT1, ssa2Δ::kanMX	this study
CHY407	ssa1Δ ssa2Δ btn2Δ	ssa1Δ::hphNT1, ssa2Δ::kanMX, btn2Δ::natNT2	this study
	ssa1Δ ssa2Δ hsp42Δ	ssa1Δ::hphNT1, ssa2Δ::kanMX, hsp42Δ::natNT2	this study
CHY486	fes1Δ sse1Δ	fes1Δ::his3MX6, sse1Δ::hphNT1	this study
CHY488	fes1Δ sse1Δ btn2Δ	fes1Δ::his3MX6, sse1Δ::hphNT1, btn2Δ::natNT2	this study
	fes1Δ sse1Δ hsp42Δ	fes1Δ::his3MX6, sse1Δ::hphNT1, hsp42Δ::natNT2	this study
CHY490	hsp104Δ sse1Δ	hsp104Δ::loxP_URA3_loxP, sse1Δ::hphNT1	this study
CHY492	hsp104Δ sse1Δ btn2Δ	hsp104Δ::loxP_URA3_loxP, btn2Δ::natNT2, sse1Δ::hphNT1	this study
	hsp104Δ sse1Δ hsp42Δ	hsp104Δ::loxP_URA3_loxP, hsp42Δ::natNT2, sse1Δ::hphNT1	this study
CHY346	btn2Δ	btn2Δ::natNT2	this study
CHY348	btn2Δ fes1Δ	btn2Δ::natNT2, fes1Δ::his3MX6	this study
CHY334	hsp104Δ btn2Δ	hsp104Δ::loxP_URA3_loxP, btn2Δ::natNT2	this study
JW49	pdr5Δ hsp42Δ	pdr5Δ::natNT2, hsp42Δ::kanMX	this study
CHY442	hsp104Δ fes1Δ hsp26Δ	hsp104Δ::loxP_URA3_loxP, fes1Δ::hphNT1, hsp26Δ::natNT1	this study
CHY361	hsp104Δ fes1Δ	hsp104Δ::loxP_URA3_loxP, fes1Δ::hphNT1, pRS415GPD	this study
CHY362	hsp104Δ fes1Δ OE-Ssa1	hsp104Δ::loxP_URA3_loxP, fes1Δ::hphNT1, pRS415GPD-Ssa1	this study
CHY364	hsp104Δ fes1Δ btn2Δ	hsp104Δ::loxP_URA3_loxP, btn2Δ::natNT2, fes1Δ::hphNT1, pRS415GPD	this study

CHY365	hsp104Δ fes1Δ btn2Δ OE-Ssa1	hsp104Δ::loxP_URA3_loxP, btn2Δ::natNT2, fes1Δ::hphNT1, pRS415GPD-Ssa1	this study
TG205	wt Nic96-mKate2 GFP-VHL	BY4741, Nic96-mKate::his3MX6, pESC-LEU-GFP-VHL	this study
TG211	hsp104Δ fes1Δ Nic96-Kate2 GFP-VHL	hsp104Δ::loxP_URA3_loxP, fes1Δ::hphNT1, Nic96-mKate::his3MX6, pESC-LEU-GFP-VHL	this study
TG248	hsp104Δ fes1Δ btn2Δ Nic96-Kate2 GFP-VHL	hsp104Δ::loxP_URA3_loxP, btn2Δ::natNT2, fes1Δ::hphNT1, Nic96-mKate::his3MX6, pESC-LEU-GFP-VHL	this study
TG212	hsp104Δ fes1Δ hsp42Δ Nic96-Kate2 GFP-VHL	hsp104Δ::loxP_URA3_loxP, fes1Δ::hphNT1, hsp42Δ::kanMX, Nic96-mKate::his3MX6, pESC-LEU-GFP-VHL	this study
TG214	hsp104Δ fes1Δ btn2Δ hsp42Δ Nic96-Kate2 GFP-VHL	hsp104Δ::loxP_URA3_loxP, btn2Δ::natNT2, fes1Δ::hphNT1, hsp42Δ::kanMX, Nic96-mKate::his3MX6, pESC-LEU-GFP-VHL	this study
CHY475	wt GFP-LuciDM-NLS	BY4741, pADH-GFP-LuciDM-NLS::His3	this study
CHY477	hsp104Δ fes1Δ GFP-LuciDM-NLS	hsp104Δ::loxP_URA3_loxP, fes1Δ::hphNT1, pADH-GFP-LuciDM-NLS::His3	this study
CHY478	hsp104Δ fes1Δ btn2Δ GFP-LuciDM-NLS	hsp104Δ::loxP_URA3_loxP, btn2Δ::natNT2, fes1Δ::hphNT1, pADH-GFP-LuciDM-NLS::His3	this study
CHY479	hsp104Δ fes1Δ hsp42Δ GFP-LuciDM-NLS	hsp104Δ::loxP_URA3_loxP, fes1Δ::hphNT1, hsp42Δ::kanMX, pADH-GFP-LuciDM-NLS::His3	this study
CHY373	hsp104Δ fes1Δ Btn2ΔNLS	hsp104Δ::loxP_URA3_loxP, fes1Δ::his3MX6, Btn2ΔNLS::hphNT1	this study
CHY371	hsp104Δ fes1Δ btn2Δ HSP42	hsp104Δ::loxP_URA3_loxP, btn2Δ::natNT2, fes1Δ::hphNT1, Hsp42::Leu2	this study
CHY372	hsp104Δ fes1Δ btn2Δ HSP42-NLS	hsp104Δ::loxP_URA3_loxP, btn2Δ::natNT2, fes1Δ::hphNT1,	this study

		Hsp42::Leu2-NLS	
CHY399	hsp104Δ fes1Δ GFP-VHL	hsp104Δ::loxP_URA3_loxP, fes1Δ::hphNT1, pESC-LEU-GFP-VHL	this study
CHY400	hsp104Δ fes1Δ btn2Δ GFP-VHL	hsp104Δ::loxP_URA3_loxP, btn2Δ::natNT2, fes1Δ::hphNT1, pESC-LEU-GFP-VHL	this study
CHY473	hsp104Δ fes1Δ Btn2ΔNLS GFP-VHL	hsp104Δ::loxP_URA3_loxP, fes1Δ::his3MX6, Btn2ΔNLS::hphNT1, pESC-LEU-GFP-VHL	this study
CHY474	hsp104Δ fes1Δ btn2Δ HSP42-NLS mCherry-VHL	hsp104Δ::loxP_URA3_loxP, btn2Δ::natNT2, fes1Δ::hphNT1, Hsp42::Leu2-NLS, pESC-URA-mCherry-VHL	this study
CRY078	hsp104Δ fes1Δ btn2Δ ΔPrLD-Hsp42-NLS	hsp104Δ::klURA3, btn2Δ::natNT2, fes1Δ::hphNT1 ΔPrLD-Hsp42-NLS::His3	this study
CRY080	hsp104Δ fes1Δ btn2Δ ΔPrLD-Hsp42-NLS GFP-VHL	hsp104Δ::klURA3, btn2Δ::natNT2, fes1Δ::hphNT1 ΔPrLD-Hsp42-NLS::His3 pESC-Leu-GFP-VHL	this study
CRY082	hsp104Δ fes1Δ btn2Δ GFP-VHL	hsp104Δ::klURA3, btn2Δ::natNT2, fes1Δ::hphNT1 ΔPrLD-Hsp42-NLS::His3 pESC-Leu-GFP-VHL	this study
R1158	Sis1-TetOff	pSIS1::kanR-tet07-TATA URA3::CMV-tTA MATa his3-1 leu2-0 met15-0	Open Biosystems
MK25	Sis1-TetOff, hsp42Δ	pSIS1::kanR-tet07-TATA URA3::CMV-tTA MATa his3-1 leu2-0 met15-0 hsp42Δ::hphNT1	Miller, 2015
CRY062	Sis1-TetOff, btn2Δ	R1158 pSIS1::kanR-tet07-TATA URA3::CMV-tTA, btn2Δ::his3MX6	this study
CRY058	Sis1-TetOff, hsp42Δ, btn2Δ	pSIS1::kanR-tet07-TATA URA3::CMV-tTA MATa his3-1 leu2-0 met15-0 hsp42Δ::hphNT1, btn2Δ::his3MX6	this study
CRY076	hsp42Δ, btn2Δ	hsp42Δ::KanMX, btn2Δ::his3MX6	this study

CAY1116	hsp42Δ	hsp42Δ::KanMX4	(Specht, Miller et al., 2011)
	hsp104Δ fes1Δ btn2Δ HSP26	hsp104Δ::loxP_URA3_loxP, btn2Δ::natNT2, fes1Δ::hphNT1, Hsp26::Leu2	this study
	hsp104Δ fes1Δ btn2Δ HSP26-NLS	hsp104Δ::loxP_URA3_loxP, btn2Δ::natNT2, fes1Δ::hphNT1, Hsp26::Leu2-NLS	this study
AJ0272	hsp104Δ fes1Δ btn2Δ Hsp42-NLS GFP-VHL	hsp104Δ::loxP_URA3_loxP, btn2Δ::natNT2, Hsp42-NLS:Leu2, pRS303-His3-GPD-GFP-VHL	this study
AJ0268	hsp104Δ fes1Δ btn2Δ HSP26 GFP-VHL	hsp104Δ::loxP_URA3_loxP, btn2Δ::natNT2, fes1Δ::hphNT1, Hsp26::Leu2, pRS303-His3-GPD-GFP-VHL	this study
AJ0270	hsp104Δ fes1Δ btn2Δ HSP26-NLS GFP-VHL	hsp104Δ::loxP_URA3_loxP, btn2Δ::natNT2, fes1Δ::hphNT1, Hsp26::Leu2-NLS, pRS303-His3-GPD-GFP-VHL	this study
AJ0153	wild type PHSE-GFP	pbtn2-yeGFP::Leu2	this study
AJ0155	hsp104Δ fes1Δ PHSE-GFP	hsp104Δ::loxP_URA3_loxP, fes1Δ::hphNT1, pbtn2-yeGFP::Leu2	this study
AJ0157	hsp104Δ fes1Δ btn2Δ PHSE-GFP	hsp104Δ::loxP_URA3_loxP, btn2Δ::natNT2, fes1Δ::hphNT1, pbtn2-yeGFP::Leu2	this study
AJ0274	wild type GFP-VHL	pRS303-His3-GPD-GFP-VHL	this study
AJ0276	hsp104Δ sse1Δ GFP-VHL	hsp104Δ::loxP_URA3_loxP, sse1Δ::hphNT1, pRS303-His3-GPD-GFP-VHL	this study
AJ0278	hsp104Δ sse1Δ btn2Δ GFP-VHL	hsp104Δ::loxP_URA3_loxP, btn2Δ::natNT2, sse1Δ::hphNT1, pRS303-His3-GPD-GFP-VHL	this study
AJ0280	hsp104Δ sse1Δ hsp42Δ GFP-VHL	hsp104Δ::loxP_URA3_loxP, hsp42Δ::natNT2, sse1Δ::hphNT1, pRS303-His3-GPD-GFP-VHL	this study
ORS20	hsp42Δ Btn2-flag mCherry-VHL	pdr5Δ::natNT2, hsp42Δ::kanMX, Btn2-flag, pESC-mCherry-hVHL	this study
CHY369	Hsp104Δ fes1Δ	hsp104Δ::URA3,	this study

	Btn2Δ sse1	btn2Δ::natNT2, fes1Δ::hphNT1, Sse1::Leu2	
CHY370	Hsp104Δ fes1Δ Btn2Δ sse1-NLS	hsp104Δ::URA3, btn2Δ::natNT2, fes1Δ::hphNT1, Sse1-NLS::Leu2	this study
AJ0280	Hsp104Δ fes1Δ Btn2Δ sse1-sbd-NLS	CHY369, hsp104Δ::URA3, btn2Δ::natNT2, fes1Δ::hphNT1, Sse1-sbd-NLS::Leu2	this study
ORS25	hsp42Δ btn2Δ Btn2ΔaCLD mCherry-VHL	pdr5Δ::natNT2, hsp42Δ::kanMX, btn2Δ::hphNT1, Btn2ΔaCLD-flag, pESC-mCherry-hVHL	this study
ORS19	hsp42Δ btn2Δ Btn2ΔC mCherry-VHL	pdr5Δ::natNT2, hsp42Δ::kanMX, btn2Δ::hphNT1, Btn2ΔC-flag, pESC-mCherry-hVHL	this study
CHY391	hsp42Δ btn2Δ mCherry-VHL NLS-GFP	pdr5Δ::natNT2, hsp42Δ::kanMX, btn2::hphNT1, pESC-mCherry-VHL, p415GPD-NLS-GFP	this study
CHY392	hsp42Δ btn2Δ mCherry-VHL NLS-GFP-Btn2	pdr5Δ::natNT2, hsp42Δ::kanMX, btn2::hphNT1, pESC-mCherry-VHL, p415GPD-NLS-GFP-Btn2	this study
CHY393	hsp42Δ btn2Δ mCherry-VHL NLS-GFP-Btn2ΔN	pdr5Δ::natNT2, hsp42Δ::kanMX, btn2::hphNT1, pESC-mCherry-VHL, p415GPD-NLS-GFP-Btn2ΔN	this study
CHY395	hsp42Δ btn2Δ mCherry-VHL NLS-GFP-Btn2_aCLD	pdr5Δ::natNT2, hsp42Δ::kanMX, btn2::hphNT1, pESC-mCherry-VHL, p415GPD-NLS-GFP-Btn2_aCLD	this study
CHY396	hsp42Δ btn2Δ mCherry-VHL NLS-GFP-Btn2_CTD	pdr5Δ::natNT2, hsp42Δ::kanMX, btn2::hphNT1, pESC-mCherry-VHL, p415GPD-NLS-GFP-Btn2_CTD	this study
CHY456	hsp104Δ fes1Δ Btn2ΔN-NLS	hsp104Δ::klURA3, Btn2ΔN-NLS::hphNT1, fes1Δ::his3MX6	this study
CHY514	hsp104Δ fes1Δ Btn2ΔaCLD	hsp104Δ::klURA3, fes1Δ::hphNT1, Btn2ΔaCLD-flag::natNT2	this study

CHY515	hsp104Δ fes1Δ Btn2ΔCTD	hsp104Δ::klURA3, fes1Δ::hphNT1, Btn2ΔC-flag::natNT2	this study
CHY517	hsp104Δ fes1Δ Btn2-NLS	hsp104Δ::klURA3, fes1Δ::hphNT1, Btn2-flag-NLS::natNT2	this study
TG148	hsp42Δ Btn2-flag NLS-GFP-LuciDM	pdr5Δ::natNT2, hsp42Δ::kanMX, Btn2-flag, pRS415GPD-NLS-GFP-LuciDM	this study
TG151	hsp42Δ btn2Δ Btn2ΔN-NLS-flag NLS-GFP-LuciDM	pdr5Δ::natNT2, hsp42Δ::kanMX, Btn2ΔN-flag-NLS, pRS415GPD-NLS-GFP-LuciDM	this study
CHY424	hsp42Δ Btn2-flag Nic96-mCherry Hsp104-GFP	pdr5Δ::natNT2, hsp42Δ::kanMX, Btn2-flag, Hsp104-GFP::his3MX6, Nic96-mCherry::URA	this study
CHY425	hsp42Δ btn2Δ Btn2ΔN-NLS Nic96-mCherry Hsp104-GFP	pdr5Δ::natNT2, hsp42Δ::kanMX, Btn2ΔN-flag-NLS, Hsp104-GFP::his3MX6, Nic96-mCherry::URA	this study
CHY426	hsp42Δ Btn2-flag Nic96-mCherry Sis1-GFP	pdr5Δ::natNT2, hsp42Δ::kanMX, Btn2-flag, Sis1-GFP::his3MX6, Nic96-mCherry::URA	this study
CHY427	hsp42Δ btn2Δ Btn2ΔN-NLS Nic96-mCherry Sis1-GFP	pdr5Δ::natNT2, hsp42Δ::kanMX, Btn2ΔN-flag-NLS, Sis1-GFP::his3MX6, Nic96-mCherry::URA	this study
ORS21	hsp42Δ btn2Δ Btn2ΔN mCherry-VHL	pdr5Δ::natNT2, hsp42Δ::kanMX, btn2Δ::hphNT1, Btn2ΔN-flag, pESC-mCherry-hVHL	this study
ORS23	hsp42Δ btn2Δ Btn2ΔNLS mCherry-VHL	pdr5Δ::natNT2, hsp42Δ::kanMX, btn2Δ::hphNT1, Btn2ΔNLS-flag, pESC-mCherry-hVHL	this study
ORS24	hsp42Δ btn2Δ Btn2ΔNLS-NLS mCherry-VHL	pdr5Δ::natNT2, hsp42Δ::kanMX, btn2Δ::hphNT1, Btn2ΔNLS-flag-NLS, pESC-mCherry-hVHL	this study

Plasmids

name	source
pCH90 (pCool6-Btn2)	this study
pCH151 (pRS305-sse1)	this study
pCH152 (pRS305-sse1-NLS)	this study
pFA6a-mKate2 (HisMX6)	this study
pESC-LEU-GFP-VHL	(Miller et al., 2015)
pRS303ADH-GFP-LuciDM-NLS	this study
pCH153 (pRS305-Hsp42)	this study
pCH154 (pRS305-Hsp42-NLS)	this study
pESC-mCherry-VHL	(Miller et al., 2015)
pRS415GPD-NLS-GFP-LuciDM	this study
pOS12 (pCool6-ΔNTD-Btn2)	this study
pCH158 (pRS415GPD-Ssa1)	this study
pCR10 (pRS303 pHsp42-Hsp42Δ99FLAG-NLS-TermHsp42)	this study
pAJ19 (pRS303-His3-GPD-GFP-VHL)	this study
pRS305-pbtn2-GFP::Leu2	this study

Supplementary Table 2

Oligonucleotides used in this study

Primer	Sequence	Purpose	source
BsaI-Btn2 F	Ggccat ggtctcaggtggt ATGTTTTCCATATTCAATTCACCATG	pCH90 (pCool6-Btn2)	This study
Btn2-.-SalI R	Ggccat gtcgac TTATATCTCCTCAATAATAGAGTTTCC	pCH90 (pCool6-Btn2)	This study
SalI-P_Sse1(-548) F	ggccat gtcgac CGCTACAATAATATGTTAGCGTGTGC	pCH151 (pRS305-sse1)	This study
Sse1-NLS Fusion R	AACTTTTCTCTTTTTCTTTGGCCCACCAGAACC GTCCATGTCAACATCACCTTCAG	pCH152 (pRS305-sse1-NLS)	This study
Sse1-NLS Fusion F	GGTTCTGGTGGG CCAAAGAAAAAGAGAAAAGTT TAATGTTAATGCAGCAAAGTAACTAGAAAAGG	pCH152 (pRS305-sse1-NLS)	This study
T_Sse1(+500)-SpeI R	Ggccat actagt CGAACAGAAAGATGGAGGAGACC	pCH151 (pRS305-sse1)	This study
SalI-P_Hsp42(-470) F	ggccat gtcgac CAGGATATGACATACTTCAATTCAGC	pCH153 (pRS305-Hsp42)	This study
Hsp42-NLS Fusion R	AACTTTTCTCTTTTTCTTTGGCCCACCAGAACC ATTTTCTACCGTAGGGTTGGGATTTTC	pCH154 (pRS305-Hsp42-NLS)	This study
Hsp42-NLS Fusion F	GGTTCTGGTGGG CCAAAGAAAAAGAGAAAAGTT TGAATATCGTATCTGTTTATACACACATAC	pCH154 (pRS305-Hsp42-NLS)	This study
T_Hsp42(+500)-SpeI R	Ggccat actagt GGAGTCTTTCAAGAGAAGATGTACC	pCH153 (pRS305-Hsp42)	This study
BsaI-Btn2_101F	Ggccat ggtctcaggtggt atg CATTACAGACCAACTTACCATGTTG	pOS12 (pCool6-ΔNTDBtn2)	This study
SpeI-Ssa1 F	GGATCC actagt ATGTCAAAAGCTGTCGGTATTGATTTAGG	pCH158 (pRS415GPD-Ssa1)	This study
Ssa1-.-XhoI R	Ggccat ctcgag TTAATCAACTTCTTCAACGGTTGGACC	pCH158 (pRS415G	This study

		PD-Ssa1)	
Hsp104 -S1	CAAAGAAAAAAGAAATCAACTACACGTACC ATAAAATATACAGAATATATGCGTACGCTG CAGGTCGAC	hsp104Δ	This study
Hsp104 -S2	ATTATATTACTGATTCTTGTTCGAAAGTTT TTAAAAATCACACTATATTAAATTAATCGA TGAATTCGAGCTCG	hsp104Δ	This study
Fes1-S1	CATTACCTTTCAACGAAAGAGTAAAATAGA AAAAAAAACACATACATAACTATG CGTACGCTGCAGGTCGAC	fes1Δ	This study
Fes1-S2	GATAATATGAAATGGTGAATGTAATATCA TTTTATTTCTACGGACGTAATCAATCGATG AATTCGAGCTCG	fes1Δ	This study
Hsp42-S1	TCAGGCAATTGTCCATATCCCACACAAATT AAGATCATACCAAGCCGAAGCAATGcgtacgc tgcaggtcgac	hsp42Δ	This study
Hsp42-S2	AAATATAAATGTATGTATGTGTGTATAAAC AGATACGATATTCAATCGATGAATTCGAGC TCG	hsp42Δ	This study
S1_Btn 2	ACAACAACCAAAAGAAAATAACTAATAGAC CCCATTACAATATAGAAATG**cgtacgctgcag gtcgac**	btn2Δ	This study
S2_Btn 2	ATTTAATGCCGTAAAAATGAAAGATGGGG AGTATGTATTATCACCCATTA**atcgatgaattc gagctcg**	btn2Δ	This study
Hsp26-S1	TCTATTAAAACAGGTATCCAAAAAAGCAAA CAAACAAACTAAACAAATTAACATGcgtacg ctgcaggtcgac	hsp26Δ	This study
Hsp26-S2	GGTCCTCGCGAGAGGGACAACACTATAGAG CCAGGTCACTTTAATCGATGAATTCGAGCT CG	hsp26Δ	This study
Rpn4 S1	GTACTAATAGCTAATTTGTATCTTTTCAAA AGTTTTCTAGAATTTTCAAGCAATC ATG cgtacgctgcaggtcgac	rpn4Δ	This study
Rpn4 S2	TTTCCATTTTGTGTGAGGTTTTCTTCTTTT ATCTCCTATATAATTTGTAACCTTA atcgatgaattcgagctcg	rpn4Δ	This study
Irc25 S1	TTAACAATGACAAAAAGAATGGGCTATTTT TGTACAAAATACTGTTGCAAAT ATG cgtacgctgcaggtcgac	irc25Δ	This study
Irc25 S2	TCTTGGATATGGCAGTCATAGATAATACAA CGCATAACTAATTTATATAGAATCA atcgatgaattcgagctcg	irc25Δ	This study
Ubr1 S1	CTCACTGAAGTCCCTAATCTTTACAGGTCA CACAAATTACATAGAACATTCCAATATG cgtacgctgcaggtcgac	ubr1Δ	This study
Ubr1 S2	TGTATAAGTTTTTATATACAAATATGTCAA CTATAAAACATAGTAGAGGGCTTGAATCTA	ubr1Δ	This study

	atcgatgaattcgagctcg		
San1 S1	CCTTTTTTCCCCTTTGTTTTCTCTCATAGTC TTGTAACCTCAGCTTTTGTTCATTATG cgtacgctgcaggtcgac	san1Δ	This study
San1 S2	TGGATGACTGCCAATAGGACATATTTTCAT ATTAACATACTTCAGAAGCGGTATTGTTTA atcgatgaattcgagctcg	san1Δ	This study
Ssa1-S1	CAAGTATTACAAGAAACAAAAAATTCAAGT AAATAACAGATAATATGCGTACGCTGCAGG TCGAC	ssa1Δ	This study
Ssa1-S2	AAGACATTTTCGTTATTATCAATTGCCGCA CCAATTGGCTTAATCGATGAATTCGAGCTC G	ssa1Δ	This study
Sse1-S1	CCTCGATAGCCATAAGCAAAAAGTACATTG ACAAACAACATTTCTTTAAAAGATGcgtacgc tgcaggtcgac	sse1Δ	This study
Sse1-S2	AAATCGGAAAAAACAATAAAGATCCTTTTC TAGTTACTTTGCTGCATTAACATTAATCGA TGAATTCGAGCTCG	sse1Δ	This study
Ssa2-S1	AATTGATTAATTCCAACAGATCAAGCAGAT TTTATACAGAAATATTTATACAATGcgtacg ctgcaggtcgac	ssa2Δ	This study
Ssa2-S2	AAAGACATTTTCGTTATTATCAATTGCCGC ACCAATTGGCTTAATCGATGAATTCGAGCT CG	ssa2Δ	This study
Pdr5-S1	CTTTTAAGTTTTCGTATCCGCTCGTTCGAA AGACTTTAGACAAAAATG CGTACGCTGCAGGTCGAC	pdr5Δ	This study
Pdr5-S2	CATCTTGGTAAGTTTCTTTTCTTAACCAAA TTCAAAATTCTATTA ATCGATGAATTCGAGCTCG	pdr5Δ	This study
mKate2 Fwd (BamHI)	GCTGGATCCATGGTGAGCGAGCTG	pFA6a-mKate2 (HisMX6)	This study
mKate2 Rev (BglII)	CGAAGATCTTCATCTGTGCCCCAG	pFA6a-mKate2 (HisMX6)	This study
NIC96-S2	CATACTGATATATAGATATAAACAAAA ATATACAATATTTAAAAAAAATCGATG AATTCGAGCTCG	TG205; TG211; TG248; TG212; TG214	This study
NIC96-S3	CCAAGGGAAACGTACAGCACTTTAATT AATATAGACGTCTCTCTACGTACGCTG CAGGTCGAC	TG205; TG211; TG248; TG212; TG214	This study
pAJ023	GGCCAT ccgcgg	pRS305-	This study

	GGACTACTTTACAGGGTAATGAATATTTGG	pBtn2-GFP	
pAJ024	gctggtattacccatggtatg	pRS305-pBtn2-GFP	This study
pAJ025	GCT GTG GGA AAA ACT TAT CGA AAG	Confirm insertion in His locus	This study
pAJ027	CGA CAT CAT CTG CCC AGA TG	pRS303-GFP-VHL	This study
pAJ029	GGCCAT ACTCGAGT TCAATCTCCCATCCGTTGATG	pRS303-GFP-VHL	This study
pAJ030	ggccat gtcgac TGGTAATCACTGGGATGTTACTGGG	pRS305-Hsp26	This study
pAJ031	Ggccat actagt CTCCTGTAGGATCTTCTCTCTTCG	pRS305-Hsp26	This study
pAJ032	AACTTTTCTCTTTTTCTTTGG CCCACCAGAACC GTTACCCCACGATTCTTGAGAAG	pRS305-Hsp26-NLS	This study
pAJ033	GGTTCTGGTGGG CCAAAGAAAAAGAGAAAAGTT TAAAGTGA CCTGGCTCTATAGTGTTG	pRS305-Hsp26-NLS	This study
pAJ034	Ggccat GCGGCCGC CTCCTGTAGGATCTTCTCTCTTCG	pRS305-Hsp26-NLS	This study
pAJ035	CCGTCAATAGAATGTTTTCAGTCTC	pRS305-Hsp26-NLS	This study
pAJ038	ggccat GGATCC TTAACCCTCACTAAAGGGAAC	pRS303-GFP-VHL	This study
pAJ039	ggccat actagt atgtctaaaggtgaagaattattc	pRS303-GFP-VHL	This study
pAJ042	CTGCTGAAGAAGTTGATTTTGTTG	pRS413-sse1-sbd-NLS	This study
MutHsp42-NLS-F	CCAAAGAAAAAGAGAAAAGTTTGAggatccA TATCGTATCTGTTTATAC	pCR10	This study
MutHsp42-NLS-R	AACTTTTCTCTTTTTCTTTGGCTTGTCATC GTCGTCCTTGTAATCATTTTCTAC	pCR10	This study
S1-BTN2	ACAACAACCAAAAGAAAATAACTAATAGAC CCCATTACAATATAGAAATGcgtacgctgcagg tcgac	btn2Δ strains	This study
S2-BTN2	ATTTAATGCCGTAAAAATGAAAGATGGGG AGTATGTATTATCACCCATTAatcgatgaattc gagctcg	btn2Δ strains	This study

Supplementary Table 3

Antibodies used in the study

Western blot analysis

Antibody	Dilution	Source
α-Btn2 (rabbit)	1:5000	lab collection
α-Luciferase (rabbit)	1:10000	lab collection
α-MDH (rabbit)	1:10000	lab collection
α-Hsp42 (rabbit)	1:5000	lab collection
α-Zwf1 (rabbit)	1:50000	lab collection
α-GFP (rabbit)	1:10000	lab collection
α-mCherry (rabbit)	1:10000	lab collection
α-Ssa1 (rabbit)	1:10000	lab collection
α-Sse1 (rabbit)	1:10000	lab collection
α-Flag (mouse)	1:1000	Sigma-Aldrich
α-Hsp26 (rabbit)	1: 5000	Lab collection
Alkaline Phosphatase Goat Anti-Rabbit IgG, AP1000	1:2500- 1:10000	Vector Laboratories
Alkaline Phosphatase Goat Anti-Mouse IgG, AP2000	1:2500- 1:10000	Vector Laboratories

Immunofluorescence

Antibody	Dilution	Source
α-Btn2 (rabbit)	1:2000	lab collection
α-Sse1 (rabbit)	1:2000	lab collection
α-Nsp1 (mouse)	1:1000	EnCor Biotechnology Inc
AlexaFluor©647 goat anti-rabbit IgG (H+L)	1:1000	Invitrogen AG
goat anti-Mouse IgG (H+L) HF 647-Labeled	1:1000	AnaSpec, Inc.

References

Miller SB, Ho CT, Winkler J, Khokhrina M, Neuner A, Mohamed MY, Guilbride DL, Richter K, Lisby M, Schiebel E, Mogk A, Bukau B (2015) Compartment-specific aggregases direct distinct nuclear and cytoplasmic aggregate deposition. EMBO J 34: 778-97

Specht S, Miller SB, Mogk A, Bukau B (2011) Hsp42 is required for sequestration of protein aggregates into deposition sites in Saccharomyces cerevisiae. J Cell Biol 195: 617-29
